# Design of multi-epitope-based therapeutic vaccine candidates from HBc and HBx proteins of hepatitis B virus using reverse vaccinology and immunoinformatics approaches

**DOI:** 10.1371/journal.pone.0313269

**Published:** 2024-12-06

**Authors:** Patricia Gita Naully, Marselina Irasonia Tan, Husna Nugrahapraja, Aluicia Anita Artarini, Reza Aditama, Ernawati Arifin Giri-Rachman

**Affiliations:** 1 School of Life Science and Technology, Institut Teknologi Bandung, Bandung, West Java, Indonesia; 2 Faculty of Health Sciences and Technology, Jenderal Achmad Yani University, Cimahi, West Java, Indonesia; 3 School of Pharmacy, Institut Teknologi Bandung, Bandung, West Java, Indonesia; 4 Biochemistry and Biomolecular Engineering Research Division, Faculty of Mathematics and Natural Sciences, Institut Teknologi Bandung, Bandung, West Java, Indonesia; Cholistan University of Veterinary and Animal Sciences, PAKISTAN

## Abstract

The major problem in cases of chronic hepatitis B (CHB) is the failure of the patient’s immune response to eliminate the covalently closed circular DNA (cccDNA) minichromosome of hepatitis B virus (HBV). Epigenetic regulation involving the HBV core protein (HBc) and HBV X protein (HBx) influences the transcription and stability of the cccDNA minichromosome. The HBc and/or HBx-based therapeutic vaccines that have been developed cannot accommodate differences between HBV genotypes. This research aims to design a therapeutic vaccine candidate based on the multi-epitope of HBc and HBx using reverse vaccinology (RV) and immunoinformatics approach. HBc and HBx sequences from 10 HBV genotypes were obtained from the NCBI Entrez Protein database. Epitopes were predicted from consensus sequences, which consisted of 13,610 HBc sequences and 12,333 HBx sequences. The study identified four cytotoxic T lymphocyte epitopes, two helper T lymphocyte epitopes, and five linear B lymphocyte that met the inclusion criteria. The vaccine candidate designed using cholera toxin subunit B and pan HLA DR-binding epitope adjuvants was predicted to be safe, antigenic, stable, and has a global population coverage of 99.43%. Molecular docking and molecular dynamics simulations demonstrated that the vaccine candidate could stably bind to B cell receptor, cytotoxic T cell receptor, and TLR4 for 100 ns. Immune response simulation indicated that it can induce antibody production and the proliferation of B and T cells. It can be concluded that RV and immunoinformatics successfully facilitated the design of a multi-epitope therapeutic vaccine candidate for CHB.

## Introduction

Although the prophylactic vaccine for hepatitis B virus (HBV) is available, around 254 million people globally remain chronically infected. Indonesia and two other countries represented 50% of the global burden [1]. According to the Hepatitis Global 2024 report, one million people have died because of chronic hepatitis B virus infection (CHB). CHB has the potential to cause a high mortality because it can progress to cirrhosis and liver cancer [2]. The main problem of CHB is the persistent presence of HBV covalently closed circular DNA (cccDNA) minichromosome in hepatocytes, whose stability, conformation, and transcription can be affected by epigenetic regulation [3–6]. One of the causes of the persistent presence of HBV cccDNA minichromosome is the failure of the immune system to eliminate the cccDNA [7–10]. Excessive antigen exposure may induce T cell exhaustion and dysfunction, leading to a decrease in T cell number due to apoptosis [7, 9].

There is currently no effective treatment for CHB. Antivirals such as Nucleos(t)ide Analogues and Pegylated Interferon Alpha 2a can only suppress virus replication but cannot eliminate the cccDNA minichromosome [2, 3, 11]. Therefore, an alternative treatment is required to induce an immune response and eliminate cccDNA minichromosome in a shorter period. Therapeutic vaccine is one of the CHB treatment options. The researcher used the HBV surface protein (HBs) as the main component of the therapeutic vaccine early in its development [8, 12]. Unfortunately, in certain studies, a therapeutic vaccine based on HBs was reported to cause T-cell exhaustion, so other HBV antigens should be used in its development [13–16]. Unlike prophylactic vaccines, therapeutic vaccines are administered to patients with CHB who have been exposed to high levels of HBs antigen for a long period, leading to T-cell exhaustion. In addition, there are differences in the types of adjuvants, dosage, frequency of vaccination, and injection sites between prophylactic and therapeutic vaccines [9, 12].

On the other hand, the HBV core protein (HBc) and HBV X protein (HBx) are two HBV proteins involved in the epigenetic regulation of the cccDNA minichromosome [7–10]. HBc and HBx can interact with the histone acetyltransferase enzyme to acetylate in histones, thereby opening the configuration of the cccDNA minichromosome [4, 5, 17]. Furthermore, these proteins can impact host cell epigenetics, inhibiting host gene expression related to immunity and tumor suppressors [4, 6]. HBx increases the programmed death ligand-1 expression through the phosphatase and tensin homolog deletion on chromosome 10/β-catenin/c-Myc signaling pathway [18]. Binding between the programmed death ligand-1 and its receptor on T cells can cause apoptosis [7].

Therefore, a therapeutic vaccine based on HBc and/or HBx may induce an immune response that targets both proteins. Damage to HBc and/or HBx could lead to HBV-related epigenetic issues, such as instability of the cccDNA minichromosome structure and inhibition of cccDNA transcription [3, 4, 6]. Although the mechanism of HBc and/or HBx destruction by therapeutic vaccine-induced immune responses remains unclear, therapeutic vaccines based on HBc and/or HBx have shown promising results compared to those based on HBs [19–23]. However, the HBc and/or HBx-based therapeutic vaccines that have been developed have some limitations.

Most therapeutic vaccines based on HBc and/or HBx have not considered the diversity of HBV, which has 10 genotypes (A-J) and 42 subgenotypes [24]. Zhang et al. [25] reported several major types of mutations in HBV, including epitope cytotoxic T lymphocyte (CTL) mutations in the HBc coding region and point mutations in the HBx coding region. Therefore, differences between the virus types infecting patients and those targeted by the vaccines can lead to therapy failure. HBV has a varied geographical distribution. Genotypes A and D are found in North America, Western Europe, and India [26]. Genotypes B and C are dominant throughout Southeast Asia, including Indonesia [24]. Genotypes F and H dominate Central America [27]. The G genotype is found in France, Germany, and America [27], while genotypes E, I, and J are found only in Western Africa [27], Vietnam [28], and Japan [29], respectively. These data suggest that therapeutic vaccines developed based on a single HBV genotype may not be effective against CHB globally.

The limitation can be overcome with multi-epitope vaccines. Multi-epitope vaccines consist of several peptides derived from multiple virus types and can induce effective responses from CTLs, helper T lymphocytes (HTLs), and B cells against the targeted virus [30]. However, multi-epitope vaccines are difficult to develop with conventional approaches because they require a long time and significant funding. Vaccine development using conventional methods involves a series of complex procedures, from pathogen cultivation to determining which proteins are immunogenic [31].

Reverse Vaccinology (RV) is one strategy to address these challenges. RV is a computational approach that uses pathogen genomic and proteomic data to identify epitopes, making vaccine development faster, more accurate, and more effective [31]. The identified epitopes can be analyzed using immunoinformatics approaches, which employ computational methods to study the immune responses. Both approaches have been extensively used in developing various multi-epitope vaccines, including those for severe acute respiratory syndrome coronavirus 2 [32], human polyomavirus type 1 [33], and human immunodeficiency virus [34]. Hence, this research aims to design HBc and HBx multi-epitope-based therapeutic vaccine candidates using RV and immunoinformatics approaches.

## Material and methods

### Construction of the consensus amino acid sequence of all HBV genotypes

All HBV protein sequences were downloaded from the NCBI Entrez Protein databases on May 5, 2024 (https://www.ncbi.nlm.nih.gov/Taxonomy/Browser/wwwtax.cgi). HBc and HBx sequences for 10 HBV genotypes were searched using the Basic Local Alignment Search Tool (BLAST) with the query of HBV genotype A2 (Accession Number: KY003230). The following BLAST parameters were used: E-value < 0.05; bitscore > 50; %identity > 30; %positive > 30, and %query coverage > 50 [35]. BLAST results were manually curated to exclude partial, truncated, and recombinant HBc and HBx sequences. The HBc and HBx sequence data were aligned using the Multiple Sequence Alignment (MSA) technique on the MAFFT server (https://mafft.cbrc.jp/alignment/server/index.html) [36]. The MSA outcomes were refined and visualized using Jalview version 2.11.3.3 software [37] to obtain consensus sequences for HBc and HBx across the 10 HBV genotypes.

### T cell epitope prediction

CTL epitopes were predicted using the NetCTL 1.2 server (https://services.healthtech.dtu.dk/services/NetCTL-1.2/) based on the epitope’s ability to bind to 12 human leukocyte antigen (HLA) allele groups, including supertype A1—B62 [38]. NetCTL 1.2 results are considered more accurate because it integrates predictions of major histocompatibility complex (MHC) class I peptide binding, proteasomal C-terminal cleavage, and transport efficiency of the transporter associated with antigen presentation [38]. Additionally, CTL epitopes were predicted using artificial neural networks on the NetMHCpan 4.1 server (https://services.healthtech.dtu.dk/services/NetMHCpan-4.1/) [39]. For this prediction, a peptide size of 9 mer was chosen, and 27 HLA alleles contained in the HLA allele reference set on the Immune Epitope Data Base (IEDB) MHC-I Binding Prediction (http://tools.iedb.org/mhci/) were used. These HLA alleles provide global coverage, encompassing more than 97% of the population [40]. NetMHCpan 4.1 uses a percentage rank threshold of 0.5% for strong binders [39].

HTL epitopes were predicted using the NetMHCIIpan 4.0 server (https://services.healthtech.dtu.dk/services/NetMHCIIpan-4.0/) [41]. A peptide size of 15 mer and 27 HLA alleles from the HLA allele reference set IEDB MHC-II Binding Prediction (http://tools.iedb.org/mhcii/) were used. These HLA alleles have the potential to cover more than 99% of the population [42]. A threshold rank of 1% was used on the server for identify strong binders [41]. In addition to globally recognized HLA, the epitopes used in this study were also assessed for recognition by Indonesian alleles (S1 Table). The Indonesia allele HLA data were retrieved from the Allele*Frequencies server (http://www.allelefrequencies.net/hla6006a.asp) [43]. The epitopes were predicted to be recognized by Indonesian allele HLA using NetMHCpan 4.1 and NetMHCIIpan 4.0. servers.

### B cell epitope prediction

The linear B lymphocyte (LBL) epitopes were predicted using the ABCpred server (http://crdd.osdd.net/raghava/abcpred/) [44]. ABCpred uses a threshold of 0.51 and has a prediction accuracy of 65.93% [44]. Additionally, LBL epitopes were predicted using Bepipred Linear Epitope Prediction 2.0 on the IEDB Antibody Epitope Prediction server (http://tools.iedb.org/bcell/). This method predicts epitopes based on the amino acid physiochemical characteristics [45]. The epitopes predicted by both methods were used for further analysis.

### T cell and B cell epitope selection

CTL, HTL, and LBL epitopes were selected based on biological activity prediction and conservancy. The epitopes antigenicity was estimated using the Vaxijen v.2.0 server (https://www.ddg-pharmfac.net/vaxijen/VaxiJen/VaxiJen.html) [46]. The server was set to target viruses, with an antigenic score threshold of 0.4 [46]. Epitopes allergenicity was predicted using AllergenFP v.1.0 server (https://www.ddg-pharmfac.net/AllergenFP/index.html), which has an accuracy rate of 88.7% [47]. The ToxinPred 3.0 server (http://crdd.osdd.net/raghava/toxin) used a hybrid ET+MERCI model to estimate epitope toxicity, with a threshold of 0.38 [48]. The AIPpred server (http://211.239.150.230/AIPpred/AIPpredMethod.html) predicted the epitope’s potential to modify inflammation or anti-inflammatory peptides (AIPs) based on its dipeptide composition [49]. To analyze autoimmunity, all epitopes were compared to human peptides using the Mimicry Peptide Database server (http://proteininformatics.org/mkumar/mipepbase/) [50]. Epitope conservation was analyzed using the IEDB Epitope Conservancy Analysis server (http://tools.iedb.org/tools/conservancy/iedb_input) [51].

CTL and HTL epitopes were selected based on their immunogenicity and propensity to stimulate interferon (IFN)-γ. The IEDB Class I Immunogenicity server (http://tools.iedb.org/immunogenicity/) predicted the immunogenicity of CTL epitopes based on the fourth to sixth positions (P4-6) and aromatic side chains [52]. A positive result indicated that the epitope is immunogenic. The HTL epitope’s ability to induce IFN-γ was predicted using a motif-based and hybrid SVM methodologies on the IFNepitope server (https://webs.iiitd.edu.in/raghava/ifnepitope/run_submit-old.php) [53]. All epitopes that were antigenic, immunogenic, non-allergenic, non-autoimmune, non-toxic, anti-inflammatory, and IFN-γ inducers, with a conservancy score of more than 50%, were considered for further analysis.

### Population coverage, cluster, and epitope novelty analysis

The population coverage of CTL and HTL epitopes was analyzed for the global population and for countries with high hepatitis B infection rates, including China, India, Indonesia, Nigeria, Philippines, and Russia using the IEDB Population Coverage server (http://tools.iedb.org/tools/population/iedb_input) [51]. Additionally, epitope cluster analysis was performed using the IEDB Epitope Cluster Analysis server (http://tools.iedb.org/cluster/) to identify overlapping epitopes [54]. Overlapping epitopes were arranged to generate a consensus for further analysis. To determine whether the selected epitopes had been previously reported, their peptide sequences were checked in the IEDB database. Epitopes were considered novel if they were not detected in the database up to the analysis date on May 21, 2024.

### Construction of multi-epitope-based therapeutic vaccine candidates

The vaccine candidate included HBc and HBx epitopes, an adjuvant, and linkers. This study compared six adjuvants: β-defensin, pan HLA DR-binding epitope (PADRE), Toll-like receptor 4 (TLR4), protein ribosome 50s (L7/L12), cholera toxin subunit B (CTB), and heparin-binding hemagglutinin adhesin (HBHA). The adjuvant was added to the N-terminal part and linked to the CTL epitope using the rigid linker EAAAK. The CTL and LBL epitopes were connected with in vivo cleavable linkers, AAY and KK, sequentially. Additionally, a flexible linker, GPGPG, was used to connect HTL epitopes.

### Vaccine candidate selection

Vaccine candidates were selected by predicting antigenicity, allergenicity, autoimmunity, and toxicity, as described in the epitope selection step. Physiochemical characteristic analysis was conducted on all vaccine candidates using the Expasy ProtParam server (https://web.expasy.org/protparam/) [55]. Key analysis results, such as instability index, aliphatic index, GRAVY, and half-life, were essential for selecting vaccine candidates. A stable vaccine candidate with heat resistance had an instability index of less than 40 and an aliphatic index of more than 50. A negative GRAVY score indicated that the vaccine candidate was hydrophilic [55]. Besides, vaccine candidate were selected based on topology prediction results using the DeppTMHMM version 1.0.24 server (https://dtu.biolib.com/DeepTMHMM). This method was effective in predicting the transmembrane alfa-helix and beta-barrel protein topologies [56]. The vaccine candidate, which was antigenic, non-allergic, non-autoimmune, non-toxic, stable, heat-resistant, hydrophilic, and had a topology outside the cell, was chosen for further analysis.

### Secondary structure prediction

The secondary structure of the selected vaccine candidate was predicted using the PSIPRED 4.0 server (http://bioinf.cs.ucl.ac.uk/psipred/) [57]. The prediction was performed in three steps: generating a sequence profile using Position-Specific Iterated BLAST (PSI-BLAST), creating the initial secondary structure with feed-forward neural networks having two hidden layers based on PSI-BLAST results, and selecting the final predicted structure [57]. For comparison, the secondary structure was also predicted using the self-optimized prediction method with an alignment server (https://npsa.lyon.inserm.fr/cgi-bin/npsa_automat.pl?page=/NPSA/npsa_sopma.html) [58].

### Prediction and validation of tertiary structures

The tertiary structure of vaccine candidate was predicted using the trRosetta server (https://yanglab.qd.sdu.edu.cn/trRosetta/) [59] and AlphaFold2 (https://colab.research.google.com/github/sokrypton/ColabFold/blob/main/AlphaFold2.ipynb) [60]. The trRosetta employed a deep neural network approach to forecast inter-residue geometry, such as distance and orientation, and used the Rosetta method to a apply direct energy minimization of modified geometries as restraints to obtain 3D structures [59]. AlphaFold2 predicted the tertiary structure of a protein by integrating physical and biological protein structure knowledge and utilizing multi-sequence alignment techniques [60]. Z-scores were used to assess the quality of the modeled 3D structures on the ProSA server (https://prosa.services.came.sbg.ac.at/prosa.php) [61, 62]. Besides, the structures were validated using the ERRAT score and Ramachandran plot on the Savesv6.0 server (https://saves.mbi.ucla.edu/).

### Molecular docking analysis

Molecular docking analysis was performed between the selected epitope and various HLA molecules that can recognize the epitope, specifically HLA-A*24:02 (PDB ID: 7MJA), HLA-B*44:03 (PDB ID: 4JQX), HLA-B*08:01 (PDB ID: 7NUI), HLA-B*27:06 (PDB ID: 5DEG), HLA-DRB1*11:01 (PDB ID: 6CPN), and HLA-DPA1*20:01/DPB1*01:01 (PDB ID: 7ZAK). The same analysis was conducted on vaccine candidates and additional receptors, including the B cell receptor (BCR) (PDB ID: 5IFH), the cytotoxic T cell receptor (TCR) (PDB ID: 6FR4), TLR4 (PDB ID: 4G8A), and TLR2 (PDB ID: 2Z7X). As a control, molecular docking analysis was also performed between these receptors and HBc (PDB ID: 7OD4) and HBx (PDB ID: 5B1Z). The crystal structures of HBc, HBx, HLA molecules, and the four receptors were retrieved from the Protein Data Bank (https://www.rcsb.org/) [63].

The GalaxyPepDock server (https://galaxy.seoklab.org/cgi-bin/submit.cgi?type=PEPDOCK) was used to model the interaction between epitopes and HLA [64]. The GalaxyPepDock uses similarity-based docking to identify templates from a database of experimental structures and creates models through energy-based optimization for structural flexibility [64]. The ClusPro 2.0 server (https://cluspro.bu.edu/publications.php) was utilized to model interactions between control and vaccine candidates with four receptors [65–68]. The server uses PIPER, a program based on the Fast Fourier Transform correlation approach, during the rigid body docking stage. PIPER’s scoring function includes structure-based pairwise interaction terms, enhancing binding accuracy and producing structures closer to the native conformation [67]. The modeling results were visualized using PyMOL (http://www.pymol.org/pymol) [69]. The PRODIGY server (https://bianca.science.uu.nl/prodigy) was employed to determine the binding affinity of the vaccine candidate to the receptor at 37°C [70, 71]. The docking complex between the epitope on the vaccine candidate and the receptor was analyzed at the residue level using the PDBsum Generate server (https://www.ebi.ac.uk/thornton-srv/databases/pdbsum/Generate.html) [72] and LigPlot+ software [73].

### Molecular dynamics (MD) simulations

MD simulations between the vaccine candidate and BCR, TCR, and TLR4 were conducted using AMBER 22 software [74] with the ff14SB force field [75]. The system was solvated using TIP3P water model and neutralized with Na^+^ and Cl^-^ ions. The protein was placed 10 Å from the box boundary. After solvation, the system underwent three stages of minimization to relax the water molecules and eliminate potential collisions among the residue side chains. It was then gradually heated to 310 K and equilibrated through six stages over a total duration of 700 ps. The simulations were performed using an NPT ensemble at 310 K and 1 bar. Each molecular dynamics simulation was run for 100 ns. Root-Mean-Square Deviation (RMSD) and Root-mean-Square Fluctuation (RMSF) analyses were conducted using CPPTRAJ [76]. The binding free energy of the system was calculated for 100 frames using the MMPBSA.py module [77].

### Immune response simulation

The immune response induced by vaccine candidate was simulated using the C-ImmSim server (http://150.146.2.1/C-IMMSIM/index.php) [78]. The simulation employed a 50 μl injection volume, 462 steps, and a 10-fold injection frequency with two week intervals (42 steps), except for the 5th and 6th injections, which had a four week interval (84 steps). The vaccination schedule used in this simulation is similar to the NASVAC vaccination schedule employed in Phase III clinical trials [19]. The simulation utilized HLA molecules that could be recognized by the epitope prediction results, specifically HLA-A*24:02, HLA-A*2301, HLA-B*0801, HLA-B*4403, HLA-DRB1*07:01, and HLA-DRB1*11:01.

## Results

### Consensus amino acid sequence construction and epitope prediction

**Fig 1:**
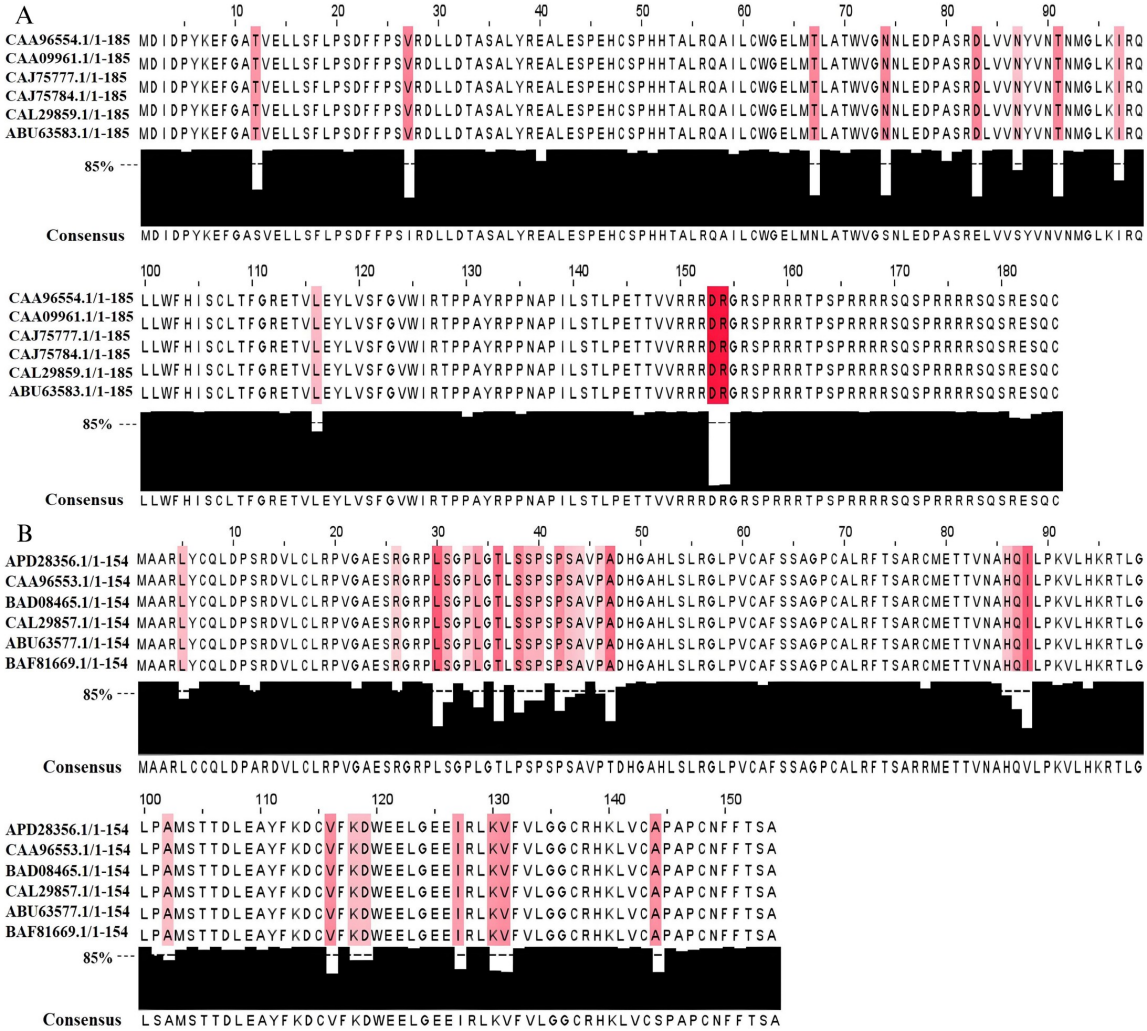
Overview of amino acid sequence alignment results from 10 hepatitis B virus (HBV) genotypes. (A) HBV core protein (HBc) consensus sequence derived from aligning 13,610 HBc sequences in the NCBI Entrez Protein database. (B) HBV X protein (HBx) consensus sequence derived from aligning 12,333 HBx sequences in the NCBI Entrez Protein database. Red highlights indicate amino acids with a conservancy rate < 85%.

The NCBI Entrez Protein database contains 19,350 HBV protein sequences. After selection using BLAST, there were 13,610 HBc sequences and 12,333 HBx sequences. Data were collected from 10 HBV genotypes across various countries in Africa, America, Australia, Asia, and Europe, spanning the years 1963 to 2023. Consensus sequences were successfully obtained using the MSA technique (Fig 1). The results showed that out of 185 amino acids in HBc and 154 amino acids in HBx, 11 amino acids in HBc and 26 amino acids in HBx have a conservation level below 85%. Consequently, there are 7 conserved regions in both HBc and HBx proteins (Table 1).

**Table 1:** Conserved regions in HBc and HBx sequences derived from 10 HBV genotypes.

Protein	Position of Peptides	Minimum Conservancy (%)	Sequences
**HBc**	1–11	96.811	MDIDPYKEFGA
	13–26	97.685	VELLSFLPSDFFPS
	28–66	88.095	RDLLDTASALYREALESPEHCSPHHTALRQAILCWGELM
	75–82	88.911	NLEDPASR
	98–115	97.046	RQLLWFHISCLTFGRETV
	117–152	93.357	EYLVSFGVWIRTPPAYRPPNAPILSTLPETTVVRRR
	155–185	90.711	GRSPRRRTPSPRRRRSQSPRRRRSQSRESQC
**HBx**	6–25	85.529	CCQLDPARDVLCLRPVGAES
	48–85	88.989	DHGAHLSLRGLPVCAFSSAGPCALRFTSARRMETTVNA
	89–101	85.843	LPKVLHKRTLGLS
	103–115	93.635	MSTTDLEAYFKDC
	120–126	95.808	WEELGEE
	132–143	95.435	FVLGGCRHKLVC
	145–154	91.430	PAPCNFFTSA

In the HBc protein, 77 CTL epitopes (S2 Table) and 15 HTL epitopes (S3 Table) were predicted to be recognized by HLA alleles from Indonesian and global populations. Additionally, four CTL epitopes (Table 2) and three HTL epitopes (Table 3) were identified as antigenic, immunogenic, non-allergenic, non-autoimmune, non-toxic, anti-inflammatory, and IFN-γ inducers. Unfortunately, only one CTL HBc epitope can be used in vaccine candidate construction because the other three epitopes rendered the vaccine candidates unstable (index instability > 40). Cluster analysis revealed that three HTL epitopes in HBc overlap, leading to the use of a single consensus peptide sequence in the development of vaccine candidates. In the HBx protein, 48 CTL epitopes and 10 HTL epitopes were predicted. Out of these, three CTL epitopes and two HTL epitopes met the inclusion standards. Since both HTL epitopes overlap, they were combined into a single consensus peptide for vaccine candidates construction.

**Table 2:** Selected CTL epitopes.

Protein	Peptide	Position of Peptides	HLA class I alleles	Antigenicity	Immunogenicity	Toxicity	Allergenicity	AIP	Autoimmunity	Conservancy (%)
**HBc**	EFGASVELL	8–16	HLA-A*24:02	0.5685	0.00615	Non-Toxin	Non-Allergen	AIP	Not trigger	59.33
**HBx**	EELGEEIRL	121–129	HLA-B*18:01, HLA-B*18:02, HLA-B*40:01, HLA-B*44:02, and HLA-B*44:03	0.4503	0.36481	Non-Toxin	Non-Allergen	AIP	Not trigger	60.47
	HLSLRGLPV	52–60	HLA-B*08:01 and HLA-B*15:02	1.588	0.0016	Non-Toxin	Non-Allergen	AIP	Not trigger	95.82
	LRGLPVCAF	55–64	HLA-B*27:06	0.4081	0.00526	Non-Toxin	Non-Allergen	AIP	Not trigger	92.7

**Table 3:** Selected HTL epitopes.

Protein	Consensus Peptide	Position of Peptides	HLA class II alleles	Antigenicity	Toxicity	Allergenicity	Induces IFN-γ	AIP	Autoimmunity	Conservancy (%)
**HBc**	ELLSFLPSDFFPSIRDL	14–30	HLA-DPA1*01:03/DPB1*02:01, HLA-DPA1*01:03/DPB1*02:02, HLA-DPA1*01:03/DPB1*04:01, HLA-DPA1*01:03/DPB1*04:02, HLA-DPA1*01:03/DPB1*23:01, HLA-DPA1*02:01/DPB1*01:01, HLA-DPA1*02:02/DPB1*02:01, and HLA-DPA1*02:02/DPB1*02:02	0.6627	Non-Toxin	Non-Allergen	Positive	AIP	Not trigger	51.61
**HBx**	AGPCALRFTSARRMETTVN	66–84	HLA-DRB1*07:01 and HLA-DRB1*11:01	0.4297	Non-Toxin	Non-Allergen	Positive	AIP	Not trigger	81.27

**Fig 2:**
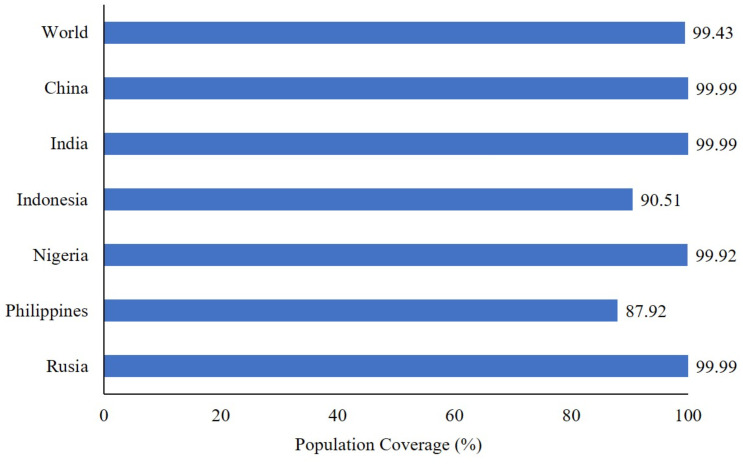
Population coverage of selected cytotoxic T lymphocyte (CTL) and helper T lymphocyte (HTL) epitopes.

**Fig 3:**
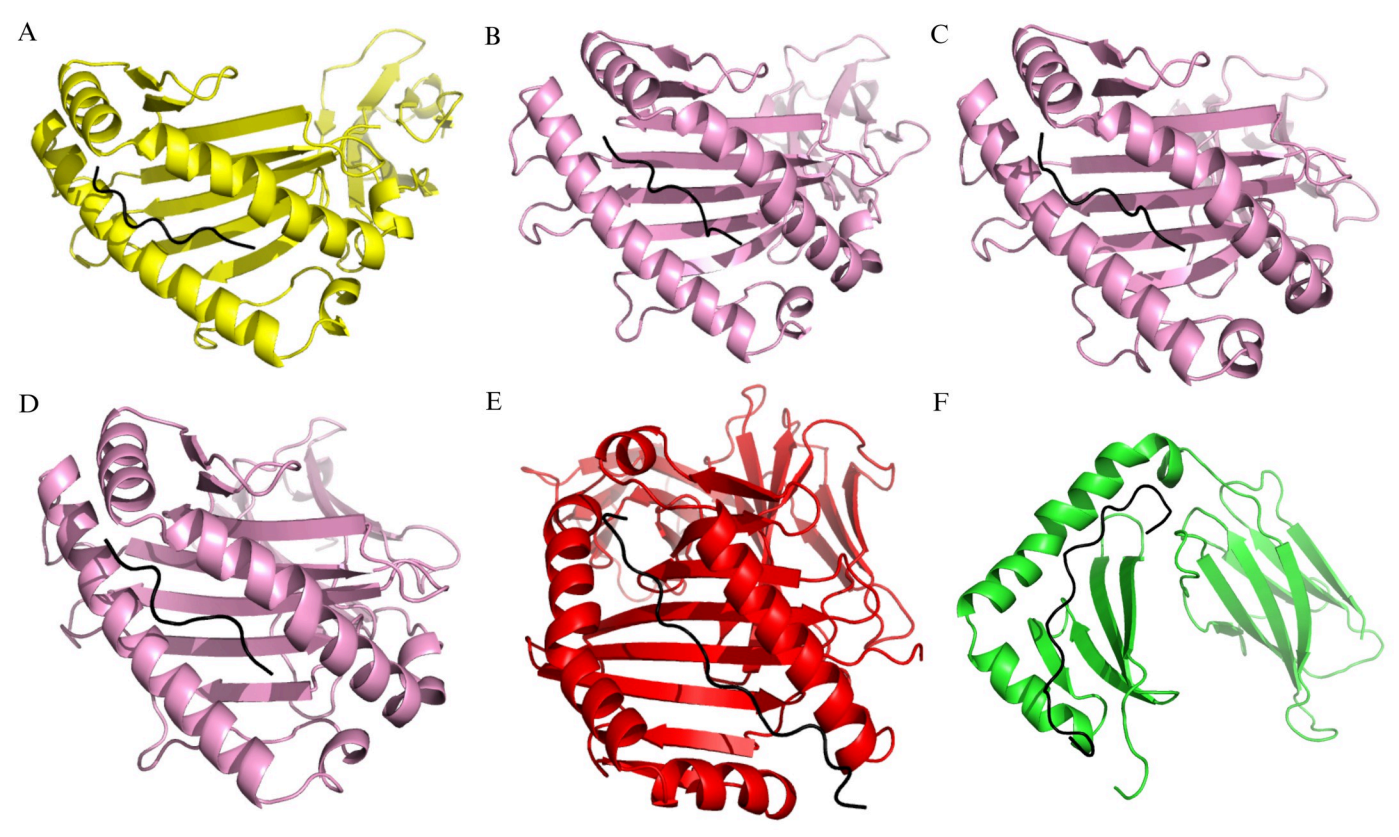
Binding pocket between epitopes and human leukocyte antigen (HLA) class I/II. (A) EFGASVELL epitope with HLA-A*24:02. (B) Epitope EELGEEIRL with HLA-B*44:03. (C) HLSLRGLPV epitope with HLA-B*08:01. (D) LRGLPVCAF epitope with HLA-B*27:06. (E) Epitope ELLSFLPSDFFPSIRDL with HLA-DPA1*02:01/DPB1*01:01 (F) Epitope AGPCALRFTSARRMETTVN with HLA-DRB1*11:01.

The selected epitopes demonstrated high population coverage in various countries with high hepatitis B prevalence, ranging from 87.92% to 99.99%, and 99.43% global population coverage (Fig 2). Molecular docking analysis indicated that these epitopes could bind to HLA class I and II global alleles (Fig 3) with energy levels of around -9.5 to -13.1 kcal/mol (Table 4). The LBL epitopes were effectively predicted using two different servers. HBc had 12 LBL epitopes, while HBx had 8 LBL epitopes (S4 Table). The selection results showed three LBL epitopes in HBc and two LBL epitopes in HBx (Table 5). All selected epitopes were singleton epitopes that met the criteria for vaccine candidate construction. Nearly all the epitopes predicted in this study were novel and had not been previously reported. Only three epitopes had been reported before, with one appearing in six references.

**Table 4:** Binding affinity energies of epitope complexes with HLA class I/II.

Epitopes	HLA class I/II alleles	ΔG (kcal/mol)	Kd (M) at 37°C
**EFGASVELL**	HLA-A*24:02	-9.7	8.2 x 10^−8^
**EELGEEIRL**	HLA-B*44:03	-9.5	1.1 x 10^−7^
**HLSLRGLPV**	HLA-B*08:01	-10.8	1.2 x 10^−8^
**LRGLPVCAF**	HLA-B*27:06	-10.7	1.4 x 10^−8^
**ELLSFLPSDFFPSIRDL**	HLA-DPA1*02:01/DPB1*01:01	-13.1	2.7 x 10^−10^
**AGPCALRFTSARRMETTVN**	HLA-DRB1*11:01	-12.1	1.4 x 10^−9^

**Table 5:** Selected LBL epitopes.

Protein	Peptides	Position of Peptides	Antigenicity	Toxicity	Allergenicity	AIP	Autoimmunity	Conservancy (%)
**HBc**	IDPYKEFGASVELLSF	3–18	0.4863	Non-Toxin	Non-Allergen	AIP	Not trigger	56.11
	HCSPHHTALRQAILCW	47–62	0.7632	Non-Toxin	Non-Allergen	AIP	Not trigger	86.9
	WFHISCLTFGRETVLE	102–117	1.5739	Non-Toxin	Non-Allergen	AIP	Not trigger	71.27
**HBx**	LSLRGLPVCAFSSAGP	52–60	0.7251	Non-Toxin	Non-Allergen	AIP	Not trigger	88.58
	KVLHKRTLGLSAMSTT	91–106	0.6841	Non-Toxin	Non-Allergen	AIP	Not trigger	52.70

### Vaccine candidate construction and structural prediction

**Fig 4:**
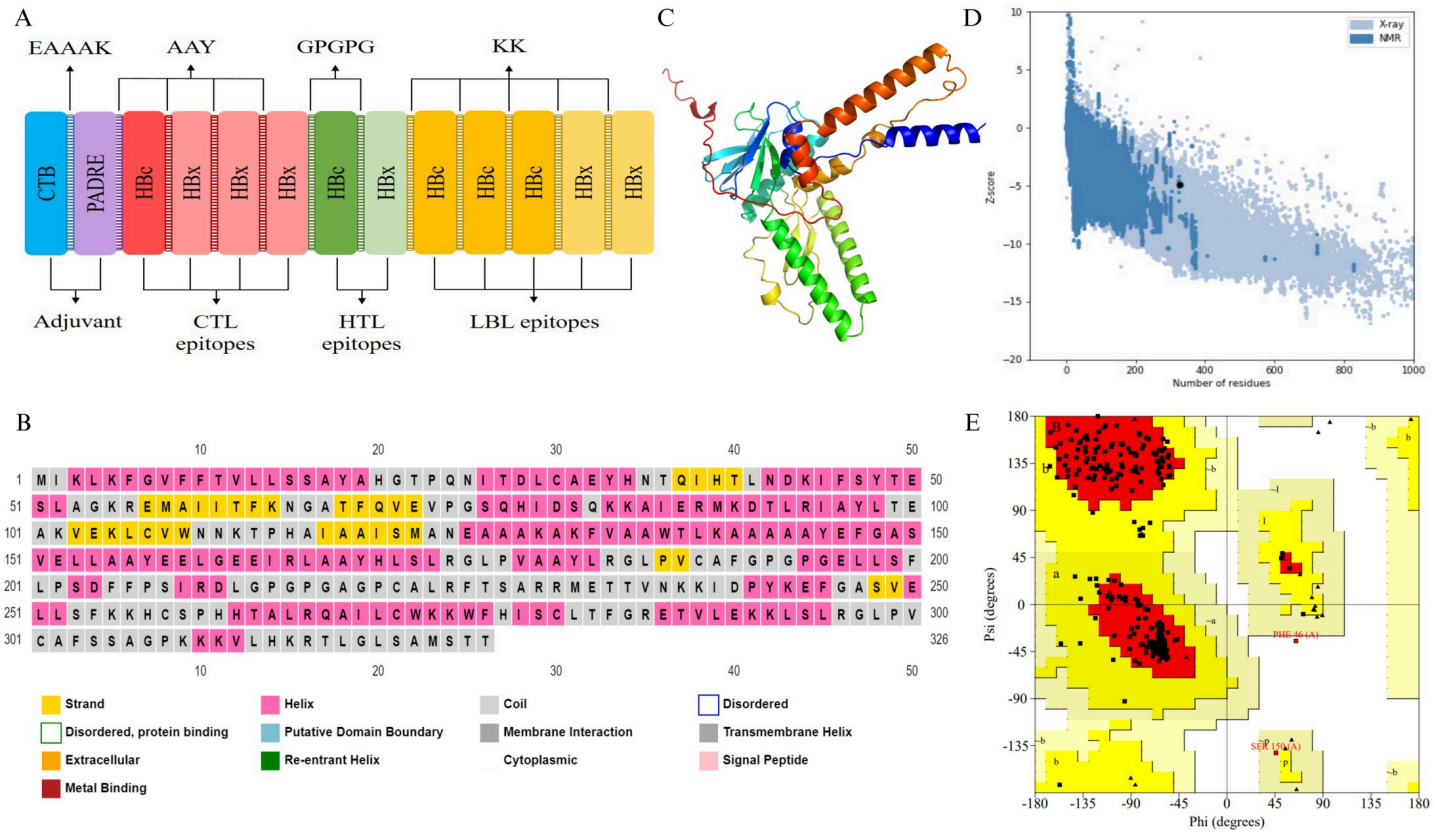
Structure prediction of the selected vaccine candidate (C10). (A) The C10 vaccine candidate consist of CTL (red), HTL (green) and LBL (yellow) epitopes, along with adjuvants. Cholera toxin subunit B (CTB) (blue) and pan HLA DR-binding epitope (PADRE) (purple) were joined using the EAAAK linker. CTL HBx epitopes, HTL epitopes, and linear B lymphocyte (LBL) epitopes were connected using AAY, GPGPG, and KK linkers, respectively. (B) The secondary structure of the C10 was predicted using PSIPRED 4.0 server. The analysis showed that the secondary structure comprises 46.01% alpha helix and 34.97% random coil. (C) The tertiary structure of the C10 was predicted using trRosetta tool. (D) The Z-score for the successfully modeled 3D structure is—4.92. (E) Validation of the 3D structure using the Ramachandran plot showed that 90.6% of amino acid residues were located in the most favored regions, and 8.7% were in the additional allowed regions.

The vaccine candidate construct included one CTL epitope from HBc, three CTL epitopes from HBx, one HTL epitope from each of HBc and HBx, three LBL epitopes from HBc, and two LBL epitopes in HBx (Fig 4A). This study evaluated six adjuvants, five of which were combined with PADRE to generate 11 vaccine candidates. The prediction results indicated that 10 of these vaccine candidates were antigenic, did not trigger allergies or autoimmune reactions, and were non-toxic (S5 Table). However, one vaccine candidate that used the 50s ribosomal protein as an adjuvant along with PADRE could potentially induce allergies, as it had a Tanimoto similarity index of 0.86 with the major allergen protein Phl p 5, which has a pollen allergen domain from the *Phleum pratense*. Physicochemical property predictions identified three stable vaccine candidates; of these, only the C10 vaccine candidate, which included the adjuvants CTB and PADRE, was hydrophilic and exhibited a topology outside the cell (Table 6).

**Table 6:** Physicochemical properties of the designed vaccine candidates.

Code	Adjuvants	Amino acid	Molecular Weight	pI	Half Life^a^ (h)	Instability	Aliphatic Index	GRAVY	Topology
**C1**	β-defensin	231	25401.88	9.71	30	47.14	84.11	-0.129	Inside
**C2**	PADRE	199	21588.31	9.55	4.4	42.12	88.89	0.07	Inside
**C3**	Ribosom 50s	313	33347.82	7.6	30	35.53	95.21	0.077	Inside
**C4**	TLR4	193	20932.47	9.41	4.4	47.33	88.08	0.01	Inside
**C5**	CTB	310	34159.77	9.31	30	41.65	87.9	-0.051	Outside
**C6**	HBHA	345	37830.24	6.38	30	46.08	90.64	-0.233	Inside
**C7**	β-defensin and PADRE	247	27036.82	9.76	30	43.47	84.66	-0.062	Inside
**C8**	Ribosom 50s and PADRE	329	34982.76	8.39	30	33.35	95.08	0.117	Inside
**C9**	TLR4 and PADRE	209	22567.41	9.53	4.4	42.99	88.42	0.078	Inside
**C10**	CTB and PADRE	326	35794.71	9.4	30	39.15	88.13	-0.004	Outside
**C11**	HBHA and PADRE	361	39465.19	7.03	30	43.62	90.72	-0.183	Inside

^a^mammalian reticulocytes, in vitro

The secondary structure of the C10 vaccine candidate was successfully predicted using two servers, with similar results. C10 consists of 150 amino acids (46.01%) forming 16 alpha-helix structures, 114 amino acids (34.97%) forming 23 coil structures, and 62 amino acids (19.02%) forming 7 β-strand structures (Fig 4B). In this study, the 3D structure of the C10 vaccine candidate predicted by the trRosetta webserver was compared with that predicted by AlphaFold2. For the subsequent analysis, we used the 3D structure predicted by trRosetta (Fig 4C), as the assessment results show that it has a Z-score of -4.92 (Fig 4D), an Errat value of 98.625%, and a Ramachandran plot value of 90.6% (Fig 4E). All three values are superior to those of the 3D structure predicted by AlphaFold2, which are -2.33 for the Z-score, 74.342% for the Errat value, and 64.1% for the Ramachandran plot value.

### Molecular docking analysis and molecular dynamics simulations

**Fig 5:**
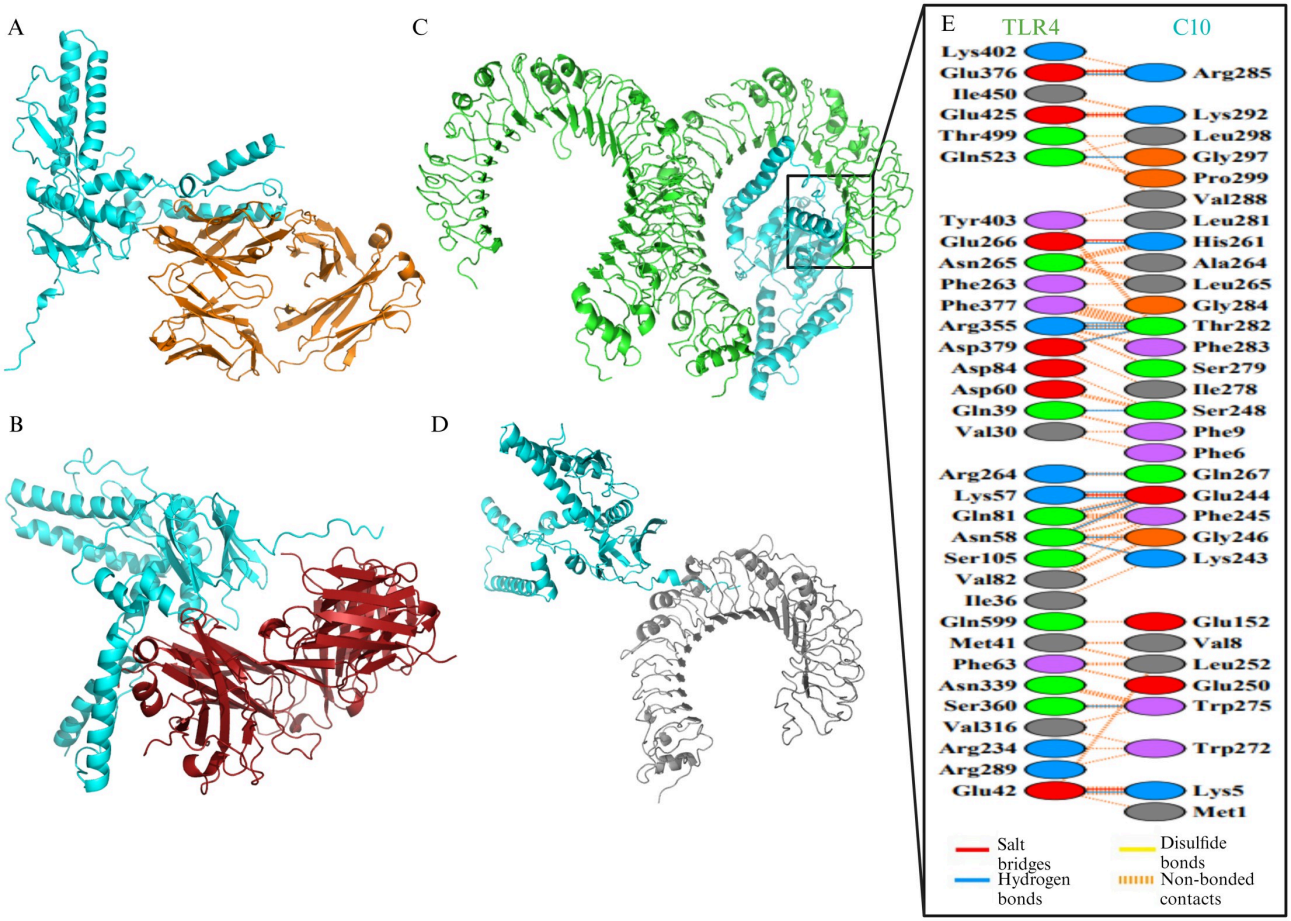
Binding mode and interaction between C10 vaccine candidate and receptors. (A) Docked complex of C10 (cyan) with B cell receptor (BCR) (orange) using molecular docking. (B) Docked complex of C10 (cyan) with cytotoxic T cell receptor (TCR) (red). (C) Docked complex of C10 (cyan) with toll-like receptor (TLR) 4 (green). (D) Docked complex of C10 (cyan) with TLR2 (gray). (E) The interaction network between vaccine candidate C10 and chain A on TLR4. The interaction involved 17 hydrogen bonds and 5 salt bridges.

**Fig 6:**
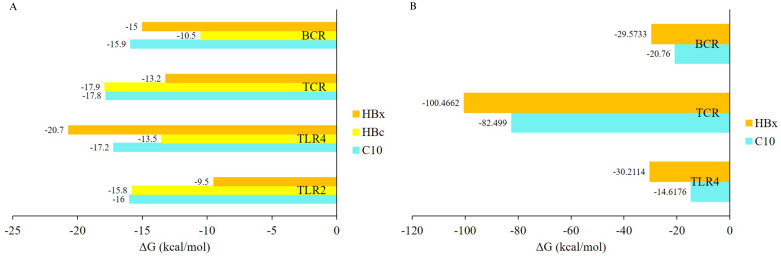
Binding free energy values between C10 vaccine candidates and receptors. (A) Binding free energy values between C10 vaccine candidate with BCR, TCR, TLR4, and TLR2 from molecular docking analysis compared to HBc and HBx proteins. (B) Binding free energy values between the C10 vaccine candidate and BCR, TCR, and TLR4 from MD simulation analysis compared to the HBx.

The modeling results of the interaction between the C10 vaccine candidate and four receptor types showed that C10 can bind to BCR (Fig 5A), TCR (Fig 5B), TLR4 (Fig 5C), and TLR2 (Fig 5D). The LigPlot+ analysis reported that the interaction between vaccine candidate C10 and TLR4 involves 17 hydrogen bonds and 3 salt bridges, while PDBsum Generate server analysis identified 17 hydrogen bonds and 5 salt bridges (Fig 5E). Although there are slight differences in the number of salt bridges, both analyses identify the same types of interactions: hydrogen bonds and salt bridges. Both tools also indicated the presence of these interactions at the same residues. LigPlot analysis revealed that salt bridges were formed between residues Glu425, Glu376, and Glu42 on TLR4 and Lys292, Arg285, and Lys5 on the vaccine candidate C10, respectively. These results are consistent with those generated by PDBsum. The free energy values for C10 interactions with BCR, TCR, TLR4, and TLR2 were -15.9 kcal/mol, -17.8 kcal/mol, -17.2 kcal/mol, and -16 kcal/mol, respectively (Fig 6A). The binding free energy of C10 with BCR and TLR2 was more negative than that of the HBc (-10.5 kcal/mol and -15.8 kcal/mol) and HBx (-15 kcal/mol and -9.5 kcal/mol). However, the binding free energy of C10 with TCR and TLR4 was still more positive than that of TCR—HBc (17.9 kcal/mol) and TLR4 –HBx (-20.7 kcal/mol).

**Fig 7:**
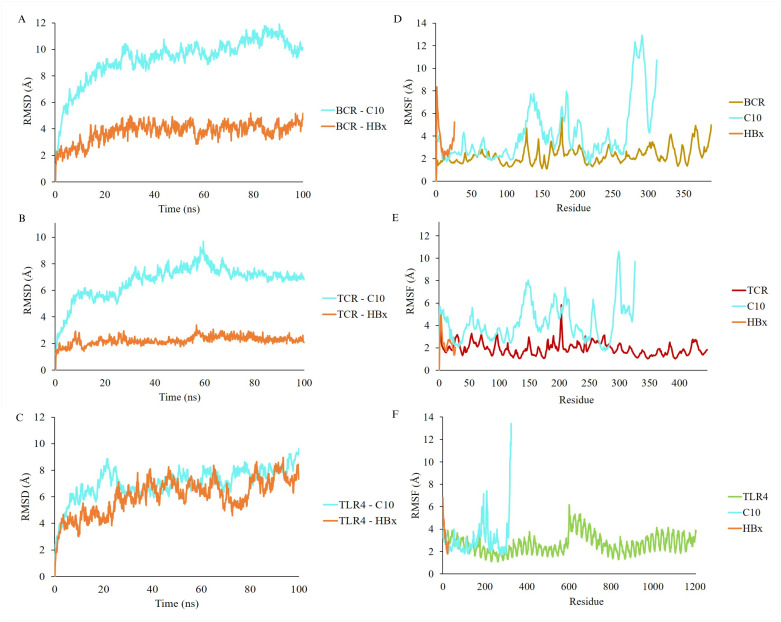
MD simulation analysis between vaccine candidate C10 and receptor using AMBER 22. (A) Root-mean-square deviation (RMSD) of BCR receptor complex with C10 (cyan) compared with HBx (orange) for 100 ns. (B) RMSD of the TCR receptor complex with C10. (C) RMSD of the TCR receptor complex with C10. (D-F) Root-mean-square fluctuation (RMSF) of BCR (gold), TCR (red), TLR4 (green) and C10 (cyan) receptors sequentially compared with HBx (orange) over 100 ns time step.

The stability of the binding between C10 and the three receptors was confirmed using molecular dynamics simulations, which included adding water to the system and increasing the temperature to 37°C. The interactions between C10 and BCR (Fig 7A), TCR (Fig 7B), and TLR4 (Fig 7C) remained stable for 100 ns, with fluctuations of about 2–4 Å. The RMSF plot, which shows the mobility of the Cα atoms during simulation, indicated that residues 291–310, 293–302, and 319–326 on C10 were flexible regions, as they exhibited higher fluctuations while binding to BCR (Fig 7D), TCR (Fig 7E), and TLR4 (Fig 7F), respectively. Additionally, molecular dynamics simulations suggested that the binding free energy of C10 with BCR, TCR, and TLR4 was more positive than that of the HBx (Fig 6B).

### Immune response simulation

**Fig 8:**
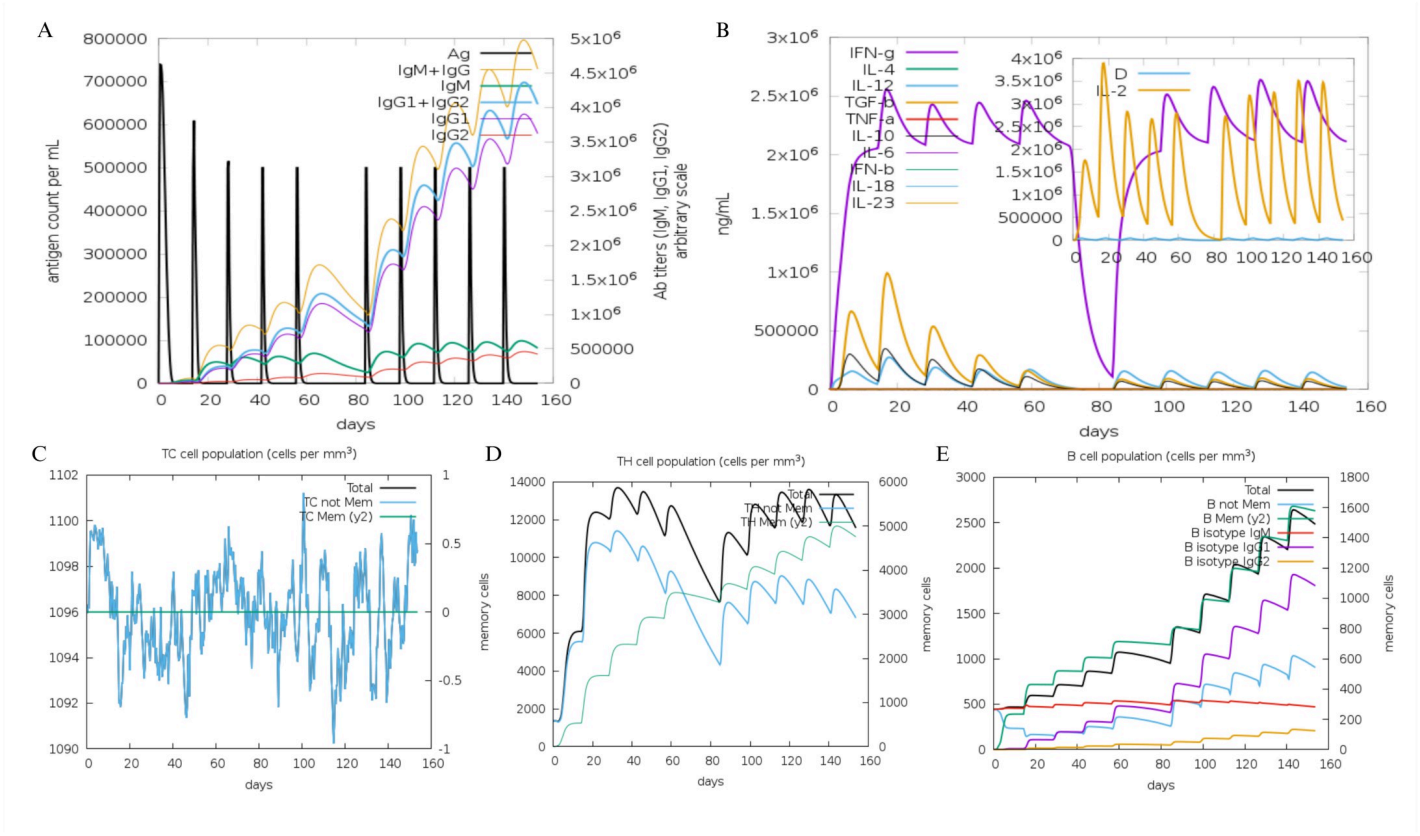
Simulation of the immune response to the C10 vaccine candidate. (A) Immunoglobulin, antigen, and immunocomplex. (B) Cytokine and interleukin concentrations. (C) Population of cytotoxic T cell. (D) Population of Helper T cell. (E) Population of B cell.

The immune system simulation indicated that the C10 vaccine candidate might have stimulate the production of IgM, IgG1, and IgG2 (Fig 8A). After 10 injections, the antibody titer (IgM and IgG) was expected to reach 4.5 x 10^6^. Furthermore, C10 was found to induce various cytokines, such as IL-2, IL-10, IL-12, IFN-γ, and TGF-β (Fig 8B). The concentration of IFN-γ produced reached 2.5x10^6^ ng/ml. Proliferation of cells involved in the immune response was also successfully induced by C10. The population of cytotoxic T cells, which target HBV in hepatocytes via non-cytolytic pathways, reached 1,100 cells/mm^3^ (Fig 8C). The populations of T helper cells (Fig 8D) and B cells (Fig 8E) also increased with the number of vaccine injections. Specifically, the population of memory T helper cells reached 5,000 cells/mm^3^ and the population of memory B cells reached 1,600 cells/mm^3^.

## Discussion

This study effectively designed multi-epitope HBc and HBx-based therapeutic vaccine candidates using the RV approach. RV refers to various computational analyses used to predict epitopes from large, continuously expanding, and easily accessible genomic or proteomic datasets [79]. To predict epitopes accurately and minimize variation between viral genotypes, consensus sequence derived from extensive datasets are required. The consensus sequences generated in this study showed several differences compared to those reported by Saeed et al. [80]. Specifically, in the HBc consensus sequence, there were variations in amino acid residues at positions 27, 67, 74, 83, 91, and 179, while the HBx consensus sequence differed at residues 36, 47, and 88. Additionally, the HBc consensus sequence generated in this study included two amino acid insertions at positions 153 and 154. The difference in consensus sequences is attributed to the data sets used: Saeed et al. [80] based their consensus sequences on 207 HBc sequences and 237 HBx sequences, whereas this study utilized 50–65 times more sequences. This difference is also reflected in the conserved regions that were successfully mapped. In this study, there were 7 conserved regions identified in both HBc and HBx, whereas Saeed et al. [80] identified 8 conserved regions in HBc and 6 conserved regions in HBx. The variaton in the amount of data used to prepare the consensus sequences likely impacts the the predicted epitopes.

Epitope prediction is a crucial aspect of RV and plays an important role in the development of vaccine candidates. Although several epitopes have been successfully predicted in HBc and HBx, these epitopes must be selected based on their biological activities. The developed vaccine candidate must be safe, which means that the epitopes should not be toxic, should not trigger allergies, and should not have similarities to human peptides to prevent autoimmune reactions. The selected epitopes must be both antigenic and immunogenic to induce an effective immune response. For combating CHB, which can lead to hepatocellular carcinoma, HTL epitopes that induce IFN-γ are necessary. IFN-γ possesses antiviral, immune-regulating, and antitumor activities [53]. It has been shown that IFN- γ generated by cytotoxic cells helps eliminate HBV through non-cytolytic mechanisms [81]. Additionally, therapeutic vaccine epitopes must be anti-inflammatory. Anti-inflammatory peptides (AIPs) are essential to prevent chronic inflammation that can lead to cancer [49].

This study successfully selected numerous epitopes that could be recognized by both Indonesian HLA and global alleles, achieving more than 90% population coverage. The selected epitopes can also be recognized by HLA-DP which has been reported to be associated with CHB [82, 83]. The majority of therapeutic vaccines only use CTL and HTL epitopes in their design because the role of B cell immunity is still not widely studied [84]. However, Zhang et al.’s research showed that LBL epitope-based therapeutic vaccines can induce antibody responses that have an impact on suppressing HBs and HBV DNA levels in the blood of test animals [85]. Consequently, this study added 5 selected LBL epitopes in the design of vaccine candidates.

All selected HBc epitopes were found in the N-terminal domain (NTD). The NTD (residues 1–140) of HBc is connected to the C-terminal domain (CTD) by a short domain [86, 87]. The NTD is responsible for capsid self-assembly, while the CTD (residues 150–183) is involved in DNA replication [86, 88]. The CTD contains 16 arginine residues that form four arginine-rich domains (ARDs) [86]. ARDs in HBc can enhance the binding of Nuclear Factor Kappa B to the HBV enhancer II region, leading to increased transcription of the *pre-core* gene [89]. Additionally, ARDs can modulate the transcriptional activator function of the host genes [87]. If the selected epitopes are part of an ARD, the constructed vaccine candidate could potentially enhance HBV replication while suppressing the expression of host genes related to immunity. In contrast, all selected HBx epitopes are found in the CTD and one epitope is located at residues 121–129. HBx also contains an NTD (residues 1–50) and CTD (residues 51–154) with two trans-activation domains, including zinc-finger motifs (residues 58–84 and 120–141), BH3-like motifs (residues 110–135) and H-box motifs (residues 88–100) [90–92]. Amino acid residues 55–60 and 121–126 are involved in regulating cccDNA transcription. Consequently, if a vaccine can stimulate the production of antibodies that target these regions, it may help inhibit HBV replication [90].

The epitope selection process was quite challenging, as only a few epitopes met the inclusion criteria and some of the selected epitopes only have a conservancy of > 50%. Many epitopes with a high level of conservation are predicted to be non-antigenic, non-immunogenic, and unsafe to use. The next challenge lies in the physicochemical properties of the epitope. In this study, physicochemical properties were not used as the basis for selection because after being analyzed, it turned out that the majority of predicted epitopes were unstable, hydrophobic, and had a topologies in the cell. There were even 3 CTL epitopes of HBc that met all inclusion criteria but could cause the 11 constructed vaccine candidates to be unstable. Therefore, the addition of adjuvants in the vaccine construction carried out in this study aims not only to enhance the immune response but also to produce a protein that is stable, hydrophilic, and has an extracellular topology. CTB is known to enhance both systemic and mucosal T-cell responses [93]. CTB can bind to the GM1-ganglysoside receptor on lymphocytes, antigen-presenting cells and epithelial cells. CTB also enhances B and T cell responses and increases antigen immunogenicity by inducing dendritic cell activation [94]. PADRE, as an adjuvant, offers several advantages, including its ability to bind to most HLA-DR molecules, its clinical safety, and its greater potency compared to other widely available HTL epitopes [95].

Besides adjuvants, linkers play a crucial role in limiting functional immunogenicity and maintaining the identity of each epitope during the vaccine processing, thereby ensuring the immunogenicity of each epitope [96]. Unlinked multi-epitope vaccines might produce new proteins with unknown properties or lead to the formation of neoepitopes [97]. The EAAAK linker is used to connect adjuvants with epitopes because it maintains a sufficient distance between them, preventing interference and allowing each component to retain its functional properties [96]. The GPGPG linker is used to link HTL epitopes due to its flexiblity and ability to induce HTL responses [98, 99]. AAY and KK linkers are used to link CTL and LBL epitopes because both can be cleaved by the proteasomes [99]. AAY specifically recognized by proteasomes in mammalian cells [100], while KK is targeted by Cathepsin B, which plays a role in antigen presentation on MHC class II molecules [101]. The combination of CTB and PADRE adjuvants, along with the use of the three linkers, significantly changed the physicochemical characteristics of the vaccine candidate.

The tertiaty structure of the C10 vaccine candidate has been successfully predicted and exhibited a favorable Z-score. A Z-score of more negative than -2 indicates good overall model quality. Z-scores that fall outside the characteristic range for native protein are considered an inappropriate structure [61, 62]. The 3D structure of C10 was also validated using the ERRAT score and Ramachandran plot. The ERRAT score assesses protein structure quality by analyzing the relative frequency of non-covalent interactions between atoms, with a score above 95% indicating a valid structure [102]. The Ramachandran plot evaluates the conformational angles of amino acid residues; a value of more that 90% in the favored region is considered optimal [103, 104].

A vaccine candidate must interact with the BCR, TCR, and TLRs to effectively induce an immune response. C10 was shown to bind to four different types of receptors through molecular docking analysis. Typically, molecular docking studies focus on interactions between vaccine candidates and TLR4, but in this study, TLR2 was also included. This is because both TLRs can detect viral proteins, leading to cytokine release [105] and inhibition of HBV replication [106]. The interaction between C10 and TLR4 formed 17 hydrogen bonds and 5 salt bridges, which was stronger than the interaction between TLR4 and HBx. There are two mutations in TLR4 used in this study; however, the results of Anwar and Choi showed that TLR4 can tolerate these mutations without affecting its structure [107]. In that study, TLR4 (PDB ID: 4G8A) was able to bind to myeloid differentiation factor-2 (MD2) and lipopolysaccharide (LPS), similar to wild-type TLR4 (PDB ID: 3FXI) [107]. Additionally, TLR4 (PDB ID: 4G8A) has a higher structural resolution of 2.4Å compared to the wild-type TLR4, which has a resolution of 3.1Å. For these reasons, TLR4 (PDB ID: 4G8A) was used in this study and several other studies [108–112].

MD simulations further confirmed the interactions of C10 with the BCR, TCR, and TLR4. The simulation showed convergence with a clear trend, despite the relatively short duration of 100 ns. The high values of RMSD and RMSF at certain residues of the vaccine candidate indicate structural flexibility. This flexibility arises as the C10 vaccine candidate and the three receptors used in the simulation adopt different conformations or undergo rearrangements to accommodate binding interactions or dynamic changes in the simulated system.

A vaccine is considered effective if it can induce an immune response. Based on the immunity simulation results, the C10 vaccine candidate was predicted to be successful in inducing antibody production and cytotoxic T-cell proliferation, both of which are important for HBV elimination. Additionally, the vaccine candidate effectively induced the production of various cytokines involved in HBV elimination. IFN-γ, produced by both adaptive and innate immune cells, can degrade cccDNA minichromosome by activating the deamination of apolipoprotein B mRNA-editing enzyme 3 [81]. In addition, IL-12 rescues exhausted cytotoxic T cells by decreasing the number of pro-apoptotic Bim molecules that can reduce the number of cytotoxic T cells [113].

RV and immunoinformatic approaches were successfully used to design the vaccine in this study, but it should be noted that both methods have limitations. RV can only identify antigens encoded by the genome, while other macromolecules, such as carbohydrates, can also act as antigens [114]. Additionally, vaccine candidates designed using RV and immunoinformatic approaches still need to be validated in the laboratory. The accuracy of predictions made through immunoinformatics depends on the quality of the data and the sophistication of the algorithms used, so these methods cannot yet replace experimental research [115]. Nevertheless, the results of this study open up opportunities for future research. The vaccine design could serve as a basis for the development of multi-epitope therapeutic vaccines with the potential to induce an immune response targeting proteins involved in the epigenetic regulation of the HBV cccDNA minichromosome.

## Conclusions

Therapeutic vaccines offer an alternative approach to addressing CHB. HBc and/or HBx-based therapeutic vaccines may induce an immune response that target proteins involved in epigenetic regulation, potentially affecting the stability and transcription of HBV cccDNA. However, existing HBc and/or HBx-based therapeutic vaccines have not accounted for differences among HBV genotypes, which may impact treatment outcomes. The study effectively used the RV technique to design vaccine candidates based on CTL, HTL, and LBL epitopes from HBc and HBx proteins across 10 different HBV genotypes. The therapeutic vaccine candidate, designed with adjuvant combination of CTB and PADRE, is predicted to be safe, antigenic, stable, and offer global population coverage of 99.43%. Additionally, the vaccine candidate can bind to four important receptors -BCR, TCR, TLR4, and TLR2- suggesting potential for inducing cytokine production and the proliferation of cytotoxic T and B cells. However, these results need to validated in the laboratory through in vitro and in vivo testing.

## Supporting information

S1 Table Indonesian HLA class I/II alleles. (DOCX)

S2 Table Predicted CTL epitopes. (DOCX)

S3 Table Predicted HTL epitopes. (DOCX)

S4 Table Predicted LBL epitopes. (DOCX)

S5 Table Biological properties of vaccine candidates. (DOCX)
