# Supplementary material for: Design of multi-epitope-based therapeutic vaccine candidates from HBc and HBx proteins of hepatitis B virus using reverse vaccinology and immunoinformatics approaches
Source: PLoS One. 2024 Dec 6;19(12):e0313269. doi: 10.1371/journal.pone.0313269 (PMC11623480; doi:10.1371/journal.pone.0313269)
Supplement: S1 Table — (DOCX) [file pone.0313269.s001.docx]

**S1 Table. Indonesian HLA class I/II alleles**

| **HLA class** | **HLA alleles** |
| --- | --- |
| I | HLA-A*01:01, HLA-A*02:01, HLA-A*02:03, HLA-A*02:06, HLA-A*02:11, HLA-A*03:01, HLA-A*11:01, HLA-A*11:04, HLA-A*24:02, HLA-A*24:07, HLA-A*24:10, HLA-A*26:01, HLA-A*29:01, HLA-A*30:01, HLA-A*32:01, HLA-A*33:03, HLA-A*34:01, HLA-A*74:01, HLA-B*07:02, HLA-B*07:05, HLA-B*08:01, HLA-B*13:01, HLA-B*13:02, HLA-B*15:01, HLA-B*15:02, HLA-B*15:10, HLA-B*15:12, HLA-B*15:13, HLA-B*15:17, HLA-B*15:21, HLA-B*15:25, HLA-B*15:32, HLA-B*18:01, HLA-B*18:02, HLA-B*27:06, HLA-B*35:01, HLA-B*35:02, HLA-B*35:03, HLA-B*35:05, HLA-B*35:30, HLA-B*37:01, HLA-B*38:02, HLA-B*39:15, HLA-B*40:01, HLA-B*40:02, HLA-B*40:06, HLA-B*41:01, HLA-B*44:03, HLA-B*48:01, HLA-B*51:01, HLA-B*51:02, HLA-B*52:01, HLA-B*56:01, HLA-B*56:02, HLA-B*56:07, HLA-B*57:01 and HLA-B*58:01 |
| II | HLA-DRB1*01:01, HLA-DRB1*03:01, HLA-DRB1*04:02, HLA-DRB1*04:03, HLA-DRB1*04:04, HLA-DRB1*08:03, HLA-DRB1*09:01, HLA-DRB1*10:01, HLA-DRB1*11:01, HLA-DRB1*11:04, HLA-DRB1*12:01, HLA-DRB1*12:02, HLA-DRB1*13:01, HLA-DRB1*13:02, HLA-DRB1*14:01, HLA-DRB1*14:04, HLA-DRB1*14:05, HLA-DRB1*14:08, HLA-DRB1*15:01, HLA-DRB1*15:02, HLA-DRB1*15:03, HLA-DRB1*16:02, HLA-DPA1*01:03/DPB1*01:01, HLA-DPA1*01:03/DPB1*02:02, HLA-DPA1*01:03/DPB1*03:01, HLA-DPA1*01:03/DPB1*04:02, HLA-DPA1*01:03/DPB1*05:01, HLA-DPA1*01:03/DPB1*13:01, HLA-DPA1*01:03/DPB1*14:01, HLA-DPA1*01:03/DPB1*23:01, HLA-DPA1*01:04/DPB1*15:01, HLA-DPA1*02:01/DPB1*02:01, HLA-DPA1*02:01/DPB1*13:01, HLA-DPA1*02:01/DPB1*17:01, HLA-DPA1*02:01/DPB1*26:01, HLA-DPA1*02:02/DPB1*01:01, HLA-DPA1*02:02/DPB1*02:01, HLA-DPA1*02:02/DPB1*02:02, HLA-DPA1*02:02/DPB1*03:01, HLA-DPA1*02:02/DPB1*04:01, HLA-DPA1*02:02/DPB1*04:02, HLA-DPA1*02:02/DPB1*05:01, HLA-DPA1*02:02/DPB1*09:01, HLA-DPA1*02:02/DPB1*13:01, HLA-DPA1*02:02/DPB1*14:01, HLA-DPA1*02:02/DPB1*17:01, HLA-DPA1*02:02/DPB1*31:01, HLA-DPA1*04:01/DPB1*03:01, HLA-DPA1*04:01/DPB1*13:01, HLA-DQA1*01:01/DQB1*05:01, HLA-DQA1*01:02/DQB1*05:01, HLA-DQA1*01:02/DQB1*05:02, HLA-DQA1*01:02/DQB1*06:02, HLA-DQA1*01:03/DQB1*06:03, HLA-DQA1*02:01/DQB1*03:01, HLA-DQA1*02:01/DQB1*03:03, HLA-DQA1*02:01/DQB1*04:02, HLA-DQA1*03:01/DQB1*03:01, HLA-DQA1*03:01/DQB1*03:02, HLA-DQA1*05:01/DQB1*02:01, HLA-DQA1*05:01/DQB1*03:01, HLA-DQA1*05:01/DQB1*03:02 and HLA-DQA1*05:01/DQB1*03:03 |
