# Supplementary material for: Design of multi-epitope-based therapeutic vaccine candidates from HBc and HBx proteins of hepatitis B virus using reverse vaccinology and immunoinformatics approaches
Source: PLoS One. 2024 Dec 6;19(12):e0313269. doi: 10.1371/journal.pone.0313269 (PMC11623480; doi:10.1371/journal.pone.0313269)
Supplement: S2 Table — (DOCX) [file pone.0313269.s002.docx]

**S2 Table.** **Predicted CTL epitopes**

| **Protein** | **Peptide** | **HLA class I alleles** | **Antigenicity** | **Immunogenicity** | **Toxicity** | **Allergenicity** | **AIP** | **Autoimmunity** | **Conservancy (%)** |
| --- | --- | --- | --- | --- | --- | --- | --- | --- | --- |
| **HBc** | ALRQAILCW | HLA-A*32:01, HLA-B*57:01, and HLA-B*15:13 | 1.3055 | 0.02276 | Non-Toxin | Allergen | AIP | Not trigger | 92.37 |
|  | APCNFFTSA | HLA-B*56:07 | -0.138 | 0.13629 | Toxin | Non-Allergen | Non-AIP | Not trigger | 86.82 |
|  | APILSTLPE | HLA-B*07:02 | 0.2201 | -0.10836 | Non-Toxin | Allergen | AIP | Not trigger | 98.58 |
|  | ASALYREAL | HLA-B*07:02, HLA-B*08:01, and HLA-B*39:01 | -0.0189 | 0.15402 | Non-Toxin | Non-Allergen | AIP | Not trigger | 84.23 |
|  | ASRELVVSY | HLA-A*01:01, HLA-A*26:01, HLA-A*29:01, HLA-A*30:01, HLA-A*30:02, HLA-A*32:01, HLA-A*34:01, HLA-B*15:01, HLA-B*15:02, HLA-B*15:12, HLA-B*15:13, HLA-B*15:17, HLA-B*15:21, HLA-B*15:25, HLA-B*15:32, HLA-B*35:01, HLA-B*35:05, HLA-B*35:30, HLA-B*57:01, and HLA-B*58:01 | 0.3647 | 0.08379 | Non-Toxin | Allergen | AIP | Not trigger | 50.75 |
|  | ASVELLSFL | HLA-A*26:01 | 0.542 | 0.02169 | Non-Toxin | Allergen | AIP | Not trigger | 59.23 |
|  | AYRPPNAPI | HLA-A*24:02, HLA-A*24:07, and HLA-A*30:01 | 0.3633 | 0.01529 | Toxin | Allergen | AIP | Not trigger | 93.1 |
|  | CSPHHTALR | HLA-A*03:01 | 0.2172 | 0.12353 | Non-Toxin | Non-Allergen | AIP | Not trigger | 92.94 |
|  | DPARDVLCL | HLA-B*56:02 | 0.3792 | 0.08438 | Toxin | Allergen | AIP | Not trigger | 80.83 |
|  | DPASRELVV | HLA-B*08:01, HLA-B*35:02, HLA-B*35:03, HLA-B*51:01, and HLA-B*51:02 | 0.1897 | 0.00564 | Non-Toxin | Non-Allergen | AIP | Not trigger | 53.97 |
|  | EFGASVELL | HLA-A*24:02 | 0.5685 | 0.00615 | Non-Toxin | Non-Allergen | AIP | Not trigger | 59.33 |
|  | EHCSPHHTA | HLA-B*15:10, HLA-B*38:02, and HLA-B*39:15 | 0.3678 | -0.11434 | Toxin | Allergen | AIP | Not trigger | 92.96 |
|  | ELMNLATWV | HLA-A*02:03 | -0.1371 | 0.1247 | Non-Toxin | Allergen | AIP | Not trigger | 54.69 |
|  | ELVVSYVNV | HLA-A*68:02 | 0.6823 | -0.07858 | Non-Toxin | Allergen | AIP | Not trigger | 50.15 |
|  | ESPEHCSPH | HLA-A*26:01 | -0.012 | -0.0682 | Toxin | Non-Allergen | AIP | Not trigger | 92.47 |
|  | EYLVSFGVW | HLA-A*23:01, HLA-A*24:02, HLA-A*24:07, and HLA-A*24:10 | 0.2567 | 0.03976 | Non-Toxin | Non-Allergen | AIP | Not trigger | 98.79 |
|  | FFPSIRDLL | HLA-A*24:02, HLA-A*24:07, and HLA-A*24:10 | 0.3253 | 0.02049 | Non-Toxin | Non-Allergen | AIP | Not trigger | 52.41 |
|  | FGRETVLEY | HLA-A*26:01, HLA-A*29:01, HLA-A*30:02, HLA-B*15:01, HLA-B*15:02, HLA-B*15:12, HLA-B*15:13, HLA-B*15:21, HLA-B*15:25, HLA-B*15:32, HLA-B*35:01, HLA-B*35:05, and HLA-B*35:30 | 0.2496 | 0.24335 | Non-Toxin | Allergen | AIP | Not trigger | 72.48 |
|  | FLPSDFFPS | HLA-A*02:06 | 0.2254 | 0.05405 | Non-Toxin | Allergen | AIP | Not trigger | 95.77 |
|  | GASVELLSF | HLA-B*15:25 and HLA-B*58:01 | 0.6411 | -0.03112 | Non-Toxin | Allergen | AIP | Not trigger | 59.29 |
|  | GELMNLATW | HLA-B*44:02 and HLA-B*44:03 | -0.2172 | -0.14134 | Non-Toxin | Allergen | AIP | Not trigger | 53.43 |
|  | GLKIRQLLW | HLA-A*32:01 | 1.5412 | -0.01056 | Non-Toxin | Allergen | AIP | Not trigger | 68.05 |
|  | GRETVLEYL | HLA-B*27:05, HLA-B*27:06, and HLA-B*38:02 | 0.0887 | 0.18366 | Non-Toxin | Allergen | AIP | Not trigger | 72.46 |
|  | GVWIRTPPA | HLA-A*02:06 | -0.1447 | 0.27692 | Toxin | Allergen | AIP | Not trigger | 90.8 |
|  | HCSPHHTAL | HLA-B*08:01, HLA-B*35:02, HLA-B*35:03, and HLA-B*39:01 | 0.6382 | 0.05271 | Toxin | Non-Allergen | AIP | Not trigger | 92.95 |
|  | HHTALRQAI | HLA-B*15:10, HLA-B*38:02, HLA-B*39:01, and HLA-B*39:15 | -0.3094 | 0.01499 | Non-Toxin | Non-Allergen | AIP | Not trigger | 91.22 |
|  | HISCLTFGR | HLA-A*03:01 and HLA-A*33:03 | 2.6885 | 0.03639 | Toxin | Non-Allergen | AIP | Not trigger | 97.39 |
|  | HTALRQAIL | HLA-B*08:01 and HLA-B*39:01 | -0.1791 | 0.05368 | Non-Toxin | Allergen | AIP | Not trigger | 92.47 |
|  | IRQLLWFHI | HLA-B*27:02 | 0.68 | 0.26665 | Non-Toxin | Allergen | AIP | Not trigger | 67.66 |
|  | KEFGASVEL | HLA-B*13:01, HLA-B*13:02, HLA-B*15:10, HLA-B*18:01, HLA-B*18:02, HLA-B*37:01, HLA-B*38:02, HLA-B*39:01, HLA-B*40:01, HLA-B*40:02, HLA-B*40:06, HLA-B*41:01, HLA-B*44:02, HLA-B*44:03, HLA-B*48:01 and HLA-B*52:01 | 0.5624 | 0.04781 | Non-Toxin | Allergen | AIP | Not trigger | 59.37 |
|  | LATWVGSNL | HLA-B*07:02 | -0.219 | 0.16419 | Non-Toxin | Allergen | AIP | Not trigger | 55.54 |
|  | LEDPASREL | HLA-B*13:01, HLA-B*15:10, HLA-B*18:01, HLA-B*18:02, HLA-B*37:01, HLA-B*38:02, HLA-B*39:01, HLA-B*39:15, HLA-B*40:01, HLA-B*40:02, HLA-B*40:06, HLA-B*41:01, HLA-B*44:02, HLA-B*44:03 and HLA-B*48:01 | -0.2917 | -0.01941 | Non-Toxin | Allergen | AIP | Not trigger | 53.83 |
|  | LESPEHCSP | HLA-B*41:01 | -0.1021 | -0.07907 | Toxin | Allergen | AIP | Not trigger | 93.45 |
|  | LEYLVSFGV | HLA-B*44:03 | 0.7605 | -0.00929 | Non-Toxin | Non-Allergen | AIP | Not trigger | 75.06 |
|  | LLDTASALY | HLA-A*01:01, HLA-A*03:01, HLA-A*29:01, HLA-A*30:02, HLA-B*15:02, HLA-B*15:02, HLA-B*15:21 and HLA-B*15:25 | 0.2347 | -0.04483 | Non-Toxin | Non-Allergen | AIP | Not trigger | 94.39 |
|  | LLSFLPSDF | HLA-B*15:25 | 0.6535 | -0.0838 | Toxin | Non-Allergen | AIP | Not trigger | 96.58 |
|  | LLWFHISCL | HLA-A*02:01 and HLA-A*02:11 | 1.6694 | 0.17536 | Non-Toxin | Non-Allergen | AIP | Not trigger | 96.55 |
|  | LPSDFFPSI | HLA-B*07:02, HLA-B*07:05, HLA-B*35:01, HLA-B*35:02, HLA-B*35:03, HLA-B*35:05, HLA-B*35:30, HLA-B*51:01, HLA-B*51:02, HLA-B*53:01 and HLA-B*56:01 | -0.0436 | 0.0868 | Non-Toxin | Allergen | AIP | Not trigger | 52.3 |
|  | LPSPSPSAV | HLA-B*56:02 and HLA-B*56:07 | 0.0124 | -0.35316 | Non-Toxin | Non-Allergen | AIP | Not trigger | 2.09 |
|  | LPVCAFSSA | HLA-B*56:07 | -0.5643 | -0.12883 | Non-Toxin | Allergen | AIP | Not trigger | 89.43 |
|  | LSFLPSDFF | HLA-B*15:17, HLA-B*15:25, and HLA_B*58:01 | 0.683 | -0.05257 | Toxin | Allergen | AIP | Not trigger | 96.46 |
|  | LTFGRETVL | HLA-B*15:17 | 0.8814 | 0.27363 | Non-Toxin | Allergen | AIP | Not trigger | 72.36 |
|  | LVSFGVWIR | HLA-A*33:03 | 0.6408 | 0.40066 | Non-Toxin | Allergen | AIP | Not trigger | 98.85 |
|  | MDIDPYKEF | HLA-B*15:02, HLA-B*18:01, HLA-B*18:02, HLA-B*37:01, HLA-B*44:02 and HLA-B*44:03 | 0.4879 | -0.07226 | Non-Toxin | Allergen | AIP | Not trigger | 95.38 |
|  | MGLKIRQLL | HLA-B*08:01 | 1.5447 | -0.14652 | Non-Toxin | Allergen | AIP | Not trigger | 67.91 |
|  | NAHQVLPKV | HLA-B*56:07 | 0.0091 | -0.21166 | Toxin | Allergen | AIP | Not trigger | 21.26 |
|  | NMGLKIRQL | HLA-B*08:01 | 2.1737 | -0.10888 | Non-Toxin | Allergen | AIP | Not trigger | 67.83 |
|  | NVNMGLKIR | HLA-A*33:03 | 2.7894 | -0.26048 | Non-Toxin | Non-Allergen | AIP | Not trigger | 51.91 |
|  | RDRGRSPRR | HLA-B*27:05 | 0.4068 | -0.03355 | Non-Toxin | Allergen | AIP | Not trigger | 10.98 |
|  | RETVLEYLV | HLA-B*37:01, HLA-B*40:01, HLA-B*40:02, HLA-B*40:06 and HLA-B*44:03 | -0.1206 | 0.12799 | Non-Toxin | Allergen | AIP | Not trigger | 72.46 |
|  | RRDRGRSPR | HLA-B*27:05 | -0.8825 | -0.0051 | Non-Toxin | Allergen | AIP | Not trigger | 10.81 |
|  | RRRRSQSPR | HLA-B*27:05 | 0.2797 | -0.34736 | Non-Toxin | Allergen | AIP | Not trigger | 97.03 |
|  | RRRRSQSRE | HLA-B*27:05 | 0.0252 | -0.31064 | Non-Toxin | Non-Allergen | AIP | Not trigger | 86.24 |
|  | RRRSQSPRR | HLA-B*27:05 | 0.5036 | -0.39732 | Non-Toxin | Non-Allergen | AIP | Not trigger | 97.23 |
|  | RRRSQSRES | HLA-B*27:05 | 0.2795 | -0.31602 | Non-Toxin | Non-Allergen | AIP | Not trigger | 83.83 |
|  | RRRTPSPRR | HLA-B*27:05 | 0.2873 | -0.08979 | Non-Toxin | Non-Allergen | AIP | Not trigger | 97.61 |
|  | RRSQSRESQ | HLA-B*27:05 | -0.1415 | -0.26788 | Non-Toxin | Allergen | AIP | Not trigger | 82.71 |
|  | RRSQSPRRR | HLA-B*27:05 | 0.1844 | -0.2948 | Non-Toxin | Allergen | AIP | Not trigger | 97.11 |
|  | RRTPSPRRR | HLA-B*27:05 | 0.6478 | -0.09618 | Non-Toxin | Allergen | AIP | Not trigger | 97.66 |
|  | RSQSPRRRR | HLA-A*03:01, HLA-A*30:01, HLA-A*31:01 and HLA-A*74:01 | 0.0978 | -0.09223 | Non-Toxin | Non-Allergen | AIP | Not trigger | 96.75 |
|  | RTPSPRRRR | HLA-A*03:01, HLA-A*30:01, HLA-A*31:01 and HLA-A*74:0 | 0.2941 | -0.05823 | Non-Toxin | Non-Allergen | AIP | Not trigger | 97.47 |
|  | SPRRRRSQS | HLA-B*07:02 and HLA-B*08:01 | 0.712 | -0.0393 | Non-Toxin | Non-Allergen | AIP | Not trigger | 98.85 |
|  | SPRRRTPSP | HLA-B*07:02, HLA-B*07:05, and HLA-B*56:01 | 1.0515 | 0.0498 | Non-Toxin | Non-Allergen | AIP | Not trigger | 96.69 |
|  | SPSPSAVPT | HLA-B*56:02 and HLA-B*56:07 | 0.5954 | -0.16077 | Non-Toxin | Non-Allergen | AIP | Not trigger | 5.62 |
|  | SRELVVSYV | HLA-B*27:02 | 0.4203 | -0.04138 | Non-Toxin | Non-Allergen | AIP | Not trigger | 50.84 |
|  | STLPETTVV | HLA-A*02:06, HLA-A*68:02 and HLA-B*52:01 | 0.3912 | 0.17616 | Non-Toxin | Allergen | AIP | Not trigger | 91.09 |
|  | SYVNVNMGL | HLA-A*23:01, HLA-A*24:02, HLA-A*24:07, HLA-A*24:10 and HLA-B*39:01 | 1.6533 | -0.0874 | Non-Toxin | Allergen | AIP | Not trigger | 51.64 |
|  | TLPETTVVR | HLA-A*33:03, HLA-A*34:01, and HLA-A*68:01 | 0.0204 | 0.23045 | Non-Toxin | Non-Allergen | AIP | Not trigger | 90.6 |
|  | TPSPRRRRS | HLA-B*07:02 | 1.2009 | 0.10818 | Non-Toxin | Non-Allergen | AIP | Not trigger | 97.34 |
|  | TVLEYLVSF | HLA-A*26:01, HLA-A*32:01, HLA-A*34:01, HLA-B*13:01, HLA-B*15:02, HLA-B*15:13, HLA-B*15:17, HLA-B*15:21, HLA-B*15:25, HLA-B*15:32, HLA-B*35:01, HLA-B*35:05 and HLA-B*35:30 | 0.0332 | 0.02129 | Non-Toxin | Allergen | AIP | Not trigger | 74.38 |
|  | VPTDHGAHL | HLA-B*56:02 and HLA-B*56:07 | 0.2231 | 0.15024 | Non-Toxin | Non-Allergen | AIP | Not trigger | 28.77 |
|  | VWIRTPPAY | HLA-A*24:02, HLA-A*29:01, HLA-A*30:02 and HLA-B*15:21 | -0.3283 | 0.13614 | Toxin | Allergen | AIP | Not trigger | 90.27 |
|  | WFHISCLTF | HLA-A*24:02 and HLA-B*15:02 | 2.0835 | -0.05411 | Non-Toxin | Allergen | AIP | Not trigger | 97.55 |
|  | WIRTPPAYR | HLA-A*31:01, HLA-A*33:03, HLA-A*34:01 and HLA-A*74:01 | -0.3559 | 0.06548 | Non-Toxin | Non-Allergen | AIP | Not trigger | 90.23 |
|  | YLVSFGVWI | HLA-A*02:01 | 0.1916 | 0.15709 | Non-Toxin | Allergen | AIP | Not trigger | 98.93 |
|  | YRPPNAPIL | HLA-B*15:10, HLA-B*27:06, HLA-B*38:02, HLA-B*39:01, HLA-B*39:15 and HLA-B*48:01 | 0.4457 | 0.08417 | Toxin | Allergen | AIP | Not trigger | 94.82 |
|  | YVNVNMGLK | HLA-A*03:01 | 1.6661 | -0.11004 | Non-Toxin | Allergen | AIP | Not trigger | 54.57 |
| **HBx** | AHQVLPKVL | HLA-B*15:10, HLA-B*38:02, HLA-B*39:01, HLA-B*39:15 and HLA-B*48:01 | -0.2495 | -0.17518 | Toxin | Non-Allergen | AIP | Not trigger | 21.28 |
|  | ALRFTSARR | HLA-A*03:01, HLA-A*31:01 and HLA-A*74:01 | 0.3033 | 0.07993 | Non-Toxin | Non-Allergen | AIP | Not trigger | 87.03 |
|  | AMSTTDLEA | HLA-A*02:01 | 1.1363 | 0.09318 | Non-Toxin | Allergen | AIP | Not trigger | 73.15 |
|  | APCNFFTSA | HLA-B*07:02 and HLA-B*56:01 | -0.138 | 0.13629 | Toxin | Non-Allergen | Non-AIP | Not trigger | 86.82 |
|  | DPARDVLCL | HLA-B*35:02, HLA-B*35:03 and HLA-B*53:01 | 0.3792 | 0.08438 | Toxin | Allergen | AIP | Not trigger | 80.83 |
|  | DPASRELVV | HLA-B*56:07 | 0.1897 | 0.00564 | Non-Toxin | Non-Allergen | AIP | Not trigger | 80.05 |
|  | EAYFKDCVF | HLA-B*08:01, HLA-B*35:01, HLA-B*35:05, HLA-B*35:30 and HLA-B*58:01 | 0.0439 | -0.0939 | Toxin | Non-Allergen | AIP | Not trigger | 51.33 |
|  | EEIRLKVFV | HLA-B*18:02, HLA-B*40:06, HLA-B*44:02 and HLA-B*44:03 | -0.1097 | -0.01528 | Non-Toxin | Allergen | AIP | Not trigger | 40.4 |
|  | EELGEEIRL | HLA-B*18:01, HLA-B*18:02, HLA-B*40:01, HLA-B*44:02 and HLA-B*44:03 | 0.4503 | 0.36481 | Non-Toxin | Non-Allergen | AIP | Not trigger | 60.47 |
|  | EIRLKVFVL | HLA-B*08:01 | 0.6393 | -0.04258 | Non-Toxin | Non-Allergen | AIP | Not trigger | 40.46 |
|  | ETTVNAHQV | HLA-A*68:02 | 0.3689 | 0.04429 | Non-Toxin | Allergen | AIP | Not trigger | 20.81 |
|  | FSSAGPCAL | HLA-B*07:02, HLA-B*15:02, HLA-B*39:01 and HLA-B*58:01 | 0.5147 | -0.01441 | Toxin | Allergen | AIP | Not trigger | 94.95 |
|  | FVLGGCRHK | HLA-A*03:01 | 0.6434 | 0.07533 | Toxin | Allergen | AIP | Not trigger | 91.62 |
|  | GAESRGRPL | HLA-B*07:02 | 1.1786 | -0.01447 | Non-Toxin | Allergen | AIP | Not trigger | 29.85 |
|  | GEEIRLKVF | HLA-B*37:01, HLA-B*44:02 and HLA-B*44:03 | 0.0823 | 0.0485 | Non-Toxin | Allergen | AIP | Not trigger | 40.53 |
|  | GPLGTLPSP | HLA-B*56:01 | -0.0511 | -0.04816 | Non-Toxin | Non-Allergen | AIP | Not trigger |  |
|  | HLSLRGLPV | HLA-B*08:01 and HLA-B*15:02 | 1.5888 | 0.0016 | Non-Toxin | Non-Allergen | AIP | Not trigger | 95.82 |
|  | KRTLGLSAM | HLA-B*27:05 and HLA-B*27:06 | 0.896 | -0.09276 | Non-Toxin | Allergen | AIP | Not trigger | 61.47 |
|  | KVFVLGGCR | HLA-A*03:01 and HLA-A*31:01 | -0.3684 | 0.09774 | Toxin | Allergen | AIP | Not trigger | 55.36 |
|  | LEAYFKDCV | HLA-B*44:02 | -0.1912 | -0.0928 | Toxin | Non-Allergen | AIP | Not trigger | 51.42 |
|  | LPKVLHKRT | HLA-B*08:01 | 0.0957 | -0.16057 | Toxin | Allergen | AIP | Not trigger | 81.79 |
|  | LPSDFFPSI | HLA-B*56:02 and HLA-B*56:07 | -0.0436 | 0.0868 | Non-Toxin | Allergen | AIP | Not trigger | 52.3 |
|  | LPSPSPSAV | HLA-B*07:02, HLA-B*07:05, HLA-B*35:01, HLA-B*35:02, HLA-B*35:03, HLA-B*35:05, HLA-B*35:30, HLA-B*51:01, HLA-B*51:02, HLA-B*53:01 and HLA-B*56:01 | 0.0124 | -0.35316 | Non-Toxin | Allergen | AIP | Not trigger | 2.09 |
|  | LPVCAFSSA | HLA-B*07:02 and HLA-B*56:01 | -0.5643 | -0.12883 | Non-Toxin | Non-Allergen | AIP | Not trigger | 89.43 |
|  | LRFTSARRM | HLA-B*27:05 and HLA-B*27:06 | 0.2093 | 0.02671 | Non-Toxin | Allergen | AIP | Not trigger | 86.55 |
|  | LRGLPVCAF | HLA-B*27:06 | 0.4081 | 0.00526 | Non-Toxin | Non-Allergen | AIP | Not trigger | 92.7 |
|  | MAARLCCQL | HLA-B*58:01 | 0.7718 | -0.10995 | Toxin | Non-Allergen | AIP | Not trigger | 66.16 |
|  | MSTTDLEAY | HLA-A*01:01, HLA-B*15:02 and HLA-B*35:01 | 0.7651 | 0.17018 | Non-Toxin | Allergen | AIP | Not trigger | 89.42 |
|  | NAHQVLPKV | HLA-A*68:02, HLA-B*51:01, HLA-B*51:02 and HLA-B*52:01 | 0.0091 | -0.21166 | Toxin | Allergen | AIP | Not trigger | 21.26 |
|  | QLDPARDVL | HLA-B*39:01 | -0.1147 | 0.1257 | Non-Toxin | Allergen | AIP | Not trigger | 81.56 |
|  | QVLPKVLHK | HLA-A*03:01, HLA-A*11:01, HLA-A*11:04, HLA-A*30:01, HLA-A*31:01, HLA-A*33:03, HLA-A*34:01, HLA-A*68:01 and HLA-A*74:01 | 0.3023 | -0.17636 | Toxin | Allergen | AIP | Not trigger | 28.74 |
|  | RGRPLSGPL | HLA-B*07:02, HLA-B*08:01 and HLA-B*15:25 | 0.3443 | -0.13877 | Toxin | Allergen | AIP | Not trigger | 6.04 |
|  | RMETTVNAH | HLA-B*15:25 | 0.359 | 0.16562 | Non-Toxin | Allergen | AIP | Not trigger | 66.52 |
|  | RPLSGPLGT | HLA-B*07:02 | -0.0144 | -0.13707 | Toxin | Non-Allergen | AIP | Not trigger | 4.29 |
|  | RRMETTVNA | HLA-B*27:05 and HLA-B*27:06 | 0.2555 | 0.14915 | Non-Toxin | Allergen | AIP | Not trigger | 83.47 |
|  | SAGPCALRF | HLA-B*58:01 | 0.6492 | 0.00505 | Non-Toxin | Allergen | AIP | Not trigger | 95.03 |
|  | SARRMETTV | HLA-B*07:02 | 0.3248 | 0.04757 | Non-Toxin | Allergen | AIP | Not trigger | 85.11 |
|  | SPSPSAVPT | HLA-B*07:02, HLA-B*07:05 and HLA-B*56:01 | 0.5954 | -0.16077 | Non-Toxin | Non-Allergen | AIP | Not trigger | 5.62 |
|  | SSAGPCALR | HLA-A*03:01, HLA-A*11:01, HLA-A*11:04, HLA-A*31:01, HLA-A*33:01, HLA-A*33:03, HLA-A*34:01, HLA-A*68:01 and HLA-A*74:01 | 0.6819 | 0.01179 | Non-Toxin | Allergen | Non-AIP | Not trigger | 95.18 |
|  | STTDLEAYF | HLA-A*26:01, HLA-B*15:02, HLA-B*15:17 and HLA-B*58:01 | 0.6384 | 0.14923 | Non-Toxin | Allergen | AIP | Not trigger | 90.27 |
|  | TDHGAHLSL | HLA-B*37:01, HLA-B*39:01, HLA-B*40:02 and HLA-B*44:03 | 0.6861 | 0.00713 | Non-Toxin | Allergen | AIP | Not trigger | 41.79 |
|  | TLPSPSPSA | HLA-A*02:01, HLA-A*02:03, HLA-A*02:06 and HLA-A*02:11 | -0.0514 | -0.44262 | Non-Toxin | Non-Allergen | AIP | Not trigger | 1.07 |
|  | TTDLEAYFK | HLA-A*01:01 and HLA-A*11:01 | 0.5932 | 0.19565 | Non-Toxin | Allergen | AIP | Not trigger | 90.49 |
|  | TTVNAHQVL | HLA-B*27:05 | 0.2887 | 0.0018 | Non-Toxin | Allergen | AIP | Not trigger | 21.18 |
|  | VCSPAPCNF | HLA-B*58:01 | -0.3892 | -0.08648 | Toxin | Allergen | AIP | Not trigger | 54.76 |
|  | VLHKRTLGL | HLA-A*02:03 and HLA-B*08:01 | 0.9917 | -0.10912 | Non-Toxin | Allergen | AIP | Not trigger | 84.76 |
|  | VLPKVLHKR | HLA-A*03:01, HLA-A*31:01, HLA-A*33:01, HLA-A*33:03 and HLA-A*74:01 | 0.323 | -0.28954 | Toxin | Non-Allergen | AIP | Not trigger | 30.66 |
|  | VPTDHGAHL | HLA-B*07:02, HLA-B*35:01, HLA-B*07:05, HLA-B*35:02, HLA-B*35:03, HLA-B*51:01 and HLA-B*53:01 | 0.0861 | 0.15024 | Non-Toxin | Non-Allergen | AIP | Not trigger | 28.77 |
