# Supplementary material for: Design of multi-epitope-based therapeutic vaccine candidates from HBc and HBx proteins of hepatitis B virus using reverse vaccinology and immunoinformatics approaches
Source: PLoS One. 2024 Dec 6;19(12):e0313269. doi: 10.1371/journal.pone.0313269 (PMC11623480; doi:10.1371/journal.pone.0313269)
Supplement: S3 Table — (DOCX) [file pone.0313269.s003.docx]

**S3 Table**. **Predicted HTL epitopes**

| **Protein** | **Peptide** | **HLA class II alleles** | **Antigenicity** | **Toxicity** | **Allergenicity** | **Induksi IFN-γ** | **AIP** | **Autoimmunity** | **Conservancy (%)** |
| --- | --- | --- | --- | --- | --- | --- | --- | --- | --- |
| HBc | ASRELVVSYVNVNMG | HLA-DRB1*15:01, HLA-DRB1*15:02 and HLA-DRB1*15:03 | 1.0105 | Non-Toxin | Allergen | Negative | Non AIP | Not trigger | 49.71 |
|  | DPASRELVVSYVNVN | HLA-DPA1*01:03/DPB1*03:01 and HLA-DPA1*01:03/DPB1*14:01 | 0.628 | Non-Toxin | Non-Allergen | Negative | Non AIP | Not trigger | 49.64 |
|  | EFGASVELLSFLPSD | HLA-DPA1*01:04/DPB1*15:01 | 0.5511 | Non-Toxin | Non-Allergen | Negative | Non AIP | Not trigger | 58.03 |
|  | ELLSFLPSDFFPSIR | HLA-DPA1*01:03/DPB1*04:02 | 0.5919 | Non-Toxin | Non-Allergen | Positive | AIP | Not trigger | 51.77 |
|  | ELVVSYVNVNMGLKI | HLA-DRB1*15:01 | 1.3588 | Non-Toxin | Allergen | Negative | Non AIP |  | 49.15 |
|  | FGASVELLSFLPSDF | HLA-DPA1*01:04/DPB1*15:01 | 0.6236 | Non-Toxin | Non-Allergen | Negative | AIP | Not trigger | 58.03 |
|  | FGVWIRTPPAYRPPN | HLA-DRB5*01:01 | 0.0484 | Non-Toxin | Non-Allergen | Positive | AIP | Not trigger | 86.26 |
|  | GASVELLSFLPSDFF | HLA-DPA1*01:04/DPB1*15:01 | 0.323 | Non-Toxin | Non-Allergen | Negative | AIP | Not trigger | 58 |
|  | GVWIRTPPAYRPPNA | HLA-DRB5*01:01 | -0.0563 | Toxin | Non-Allergen | Positive | AIP | Not trigger | 86.28 |
|  | LLSFLPSDFFPSIRD | HLA-DPA1*01:03/DPB1*02:01, HLA-DPA1*01:03/DPB1*02:02, HLA-DPA1*01:03/DPB1*04:01, HLA-DPA1*01:03/DPB1*04:02, HLA-DPA1*01:03/DPB1*23:01, HLA-DPA1*02:01/DPB1*01:01, HLA-DPA1*02:02/DPB1*02:01 and HLA-DPA1*02:02/DPB1*02:02 | 0.6538 | Non-Toxin | Non-Allergen | Positive | AIP | Not trigger | 51.57 |
|  | LSFLPSDFFPSIRDL | HLA-DPA1*01:03/DPB1*04:02 | 0.9958 | Non-Toxin | Non-Allergen | Positive | AIP | Not trigger | 51.75 |
|  | MDIDPYKEFGASVEL | HLA-DRB1*15:01 and HLA-DRB1*15:03 | 0.6777 | Non-Toxin | Non-Allergen | Negative | Non AIP | Not trigger | 56.32 |
|  | RELVVSYVNVNMGLK | HLA-DRB1*15:01, HLA-DRB1*15:02, and HLA-DRB1*15:03 | 1.3188 | Non-Toxin | Non-Allergen | Negative | Non AIP | Not trigger | 49.64 |
|  | SFGVWIRTPPAYRPP | HLA-DRB5*01:01 | 0.0167 | Non-Toxin | Non-Allergen | Positive | AIP | Not trigger | 86.24 |
|  | SRELVVSYVNVNMGL | HLA-DRB1*15:01, HLA-DRB1*15:02, and HLA-DRB1*15:03 | 1.0969 | Non-Toxin | Non-Allergen | Negative | Non AIP | Not trigger | 49.67 |
| HBx | AGPCALRFTSARRME | HLA-DRB1*07:01 | 0.5359 | Non-Toxin | Non-Allergen | Negative | Non AIP | Not trigger | 83.27 |
|  | ALRFTSARRMETTVN | HLA-DRB1*11:01 | 0.4047 | Non-Toxin | Non-Allergen | Negative | Non AIP | Not trigger | 83.5 |
|  | FTSARRMETTVNAHQ | HLA-DPA1*01:03/DPB1*03:01, HLA-DPA1*01:03/DPB1*14:01, HLA-DPA1*02:01/DPB1*14:01, HLA-DPA1*02:02/DPB1*03:01, HLA-DPA1*02:02/DPB1*14:01, and HLA-DPA1*04:01/DPB1*03:01 | 0.3638 | Non-Toxin | Non-Allergen | Negative | Non AIP | Not trigger | 37.62 |
|  | GLSAMSTTDLEAYFK | HLA-DQA1*01:03/DQB1*06:03 | 0.9247 | Non-Toxin | Non-Allergen | Negative | Non AIP | Not trigger | 60.12 |
|  | GPCALRFTSARRMET | HLA-DRB1*07:01 | 0.3505 | Non-Toxin | Allergen | Negative | Non AIP | Not trigger | 84.92 |
|  | PCALRFTSARRMETT | HLA-DRB1*07:01 | 0.2909 | Non-Toxin | Non-Allergen | Negative | Non AIP | Not trigger | 84.42 |
|  | RFTSARRMETTVNAH | HLA-DPA1*01:03/DPB1*14:01 and HLA-DPA1*0401/DPB1*0301 | 0.1241 | Non-Toxin | Non-Allergen | Negative | Non AIP | Not trigger | 65.22 |
|  | RRMETTVNAHQVLPK | DQA1*0201-DQB1*0402 | 0.1494 | Non-Toxin | Non-Allergen | Negative | Non AIP | Not trigger | 19.67 |
|  | SARRMETTVNAHQVL | HLA-DPA1*01:03/DPB1*03:01, HLA-DPA1*01:03/DPB1*14:01, and HLA-DPA1*04:01/DPB1*03:01 | 0.2255 | Non-Toxin | Non-Allergen | Negative | Non AIP | Not trigger | 19.57 |
|  | TSARRMETTVNAHQV | HLA-DPA1*01:03/DPB1*03:01, HLA-DPA1*01:03/DPB1*14:01, HLA-DPA1*02:01/DPB1*14:01, HLA-DPA1*02:02/DPB1*03:01, HLA-DPA1*02:02/DPB1*14:01, HLA-DPA1*02:01/DPB1*26:01, HLA-DPA1*04:01/DPB1*03:01, and HLA-DPA1*04:01/DPB1*13:01 | 0.367 | Non-Toxin | Non-Allergen | Negative | Non AIP | Not trigger | 19.52 |
