# Supplementary material for: Design of multi-epitope-based therapeutic vaccine candidates from HBc and HBx proteins of hepatitis B virus using reverse vaccinology and immunoinformatics approaches
Source: PLoS One. 2024 Dec 6;19(12):e0313269. doi: 10.1371/journal.pone.0313269 (PMC11623480; doi:10.1371/journal.pone.0313269)
Supplement: S4 Table — (DOCX) [file pone.0313269.s004.docx]

**S4 Table.** **Predicted LBL epitopes**

| **Protein** | **Peptide** | **Antigenicity** | **Toxicity** | **Allergenicity** | **AIP** | **Autoimmunity** | **Conservancy (%)** |
| --- | --- | --- | --- | --- | --- | --- | --- |
| **HBc** | IDPYKEFGASVELLSF | 0.4863 | Non-Toxin | Non-Allergen | AIP | Not trigger | 56.11% |
|  | FGASVELLSFLPSDFF | 0.3429 | Non-Toxin | Allergen | AIP | Not trigger | 57.91% |
|  | LPSDFFPSIRDLLDTA | 0.1361 | Non-Toxin | Allergen | AIP | Not trigger | 51.57% |
|  | SALYREALESPEHCSP | -0.1509 | Non-Toxin | Non-Allergen | AIP | Not trigger | 81.89% |
|  | HCSPHHTALRQAILCW | 0.7632 | Non-Toxin | Non-Allergen | AIP | Not trigger | 86.90% |
|  | GELMNLATWVGSNLED | 0.2177 | Non-Toxin | Allergen | AIP | Not trigger | 52.24% |
|  | VGSNLEDPASRELVVS | 0.259 | Non-Toxin | Non-Allergen | AIP | Not trigger | 50.15% |
|  | DPASRELVVSYVNVNM | 0.7033 | Non-Toxin | Non-Allergen | Non-AIP | Not trigger | 49.54% |
|  | GLKIRQLLWFHISCLT | 1.6197 | Non-Toxin | Allergen | AIP | Not trigger | 66.83% |
|  | WFHISCLTFGRETVLE | 1.5739 | Non-Toxin | Non-Allergen | AIP | Not trigger | 71.27% |
|  | TVLEYLVSFGVWIRTP | -0.0255 | Non-Toxin | Non-Allergen | AIP | Not trigger | 73.81% |
|  | GVWIRTPPAYRPPNAP | -0.0181 | Non-Toxin | Non-Allergen | AIP | Not trigger | 86.21% |
| **HBx** | TDHGAHLSLRGLPVCA | 0.9018 | Non-Toxin | Non-Allergen | AIP | Not trigger | 40.98% |
|  | LSLRGLPVCAFSSAGP | 0.7251 | Non-Toxin | Non-Allergen | AIP | Not trigger | 88.58% |
|  | SARRMETTVNAHQVLP | 0.3102 | Non-Toxin | Allergen | AIP | Not trigger | 19.55% |
|  | KVLHKRTLGLSAMSTT | 0.6841 | Non-Toxin | Non-Allergen | AIP | Not trigger | 52.70% |
|  | GLSAMSTTDLEAYFKD | 0.9195 | Non-Toxin | Allergen | AIP | Not trigger | 59.96% |
|  | TDLEAYFKDCVFKDWE | 0.1875 | Toxin | Non-Allergen | AIP | Not trigger | 29.90% |
|  | CVFKDWEELGEEIRLK | 0.7041 | Non-Toxin | Non-Allergen | AIP | Not trigger | 16.26% |
|  | LGEEIRLKVFVLGGCR | 0.2004 | Non-Toxin | Allergen | AIP | Not trigger | 38.83% |
