# Supplementary material for: Design of multi-epitope-based therapeutic vaccine candidates from HBc and HBx proteins of hepatitis B virus using reverse vaccinology and immunoinformatics approaches
Source: PLoS One. 2024 Dec 6;19(12):e0313269. doi: 10.1371/journal.pone.0313269 (PMC11623480; doi:10.1371/journal.pone.0313269)
Supplement: S5 Table — (DOCX) [file pone.0313269.s005.docx]

**S5 Table**. **Biological properties of vaccine candidates**

| **Code** | **Adjuvant** | **Antigenicity** | **Toxicity** | **Allergenicity** | **Autoimmunity** |
| --- | --- | --- | --- | --- | --- |
| **C1** | β-defensin | 0.582 | Non-Toxin | Non-Allergen | Not trigger |
| **C2** | PADRE | 0.5884 | Non-Toxin | Non-Allergen | Not trigger |
| **C3** | Ribosom 50s | 0.4959 | Non-Toxin | Non-Allergen | Not trigger |
| **C4** | TLR4 | 0.5834 | Non-Toxin | Non-Allergen | Not trigger |
| **C5** | CTB | 0.5639 | Non-Toxin | Non-Allergen | Not trigger |
| **C6** | HBHA | 0.524 | Non-Toxin | Non-Allergen | Not trigger |
| **C7** | β-defensin and PADRE | 0.5996 | Non-Toxin | Non-Allergen | Not trigger |
| **C8** | Ribosom 50s and PADRE | 0.5133 | Non-Toxin | Allergen | Not trigger |
| **C9** | TLR4 and PADRE | 0.6042 | Non-Toxin | Non-Allergen | Not trigger |
| **C10** | CTB and PADRE | 0.5781 | Non-Toxin | Non-Allergen | Not trigger |
| **C11** | HBHA and PADRE | 0.5386 | Non-Toxin | Non-Allergen | Not trigger |
